# Discovery of a novel NAMPT inhibitor that selectively targets NAPRT-deficient EMT-subtype cancer cells and alleviates chemotherapy-induced peripheral neuropathy

**DOI:** 10.7150/thno.85356

**Published:** 2023-09-11

**Authors:** Minjee Kim, Hyeyoung Kim, Bu-Gyeong Kang, Jooyoung Lee, Taegun Kim, Hwanho Lee, Jane Jung, Myung Joon Oh, Seungyoon Seo, Myung-Jeom Ryu, Yeojin Sung, Yunji Lee, Jeonghun Yeom, Gyoonhee Han, Sun-Shin Cha, Hosung Jung, Hyun Seok Kim

**Affiliations:** 1 Graduate School of Medical Science, Brain Korea 21 Project, Yonsei University College of Medicine, Seoul, 03722, Republic of Korea.; 2 Department of Biomedical Sciences, Yonsei University College of Medicine, Seoul, 03722, Republic of Korea.; 3 Department of Anatomy, Yonsei University College of Medicine, Seoul, 03722, Republic of Korea.; 4 Department of Chemistry and Nanoscience, Ewha Womans University, Seoul, 03760, Republic of Korea.; 5 Checkmate Therapeutics Inc., Seoul, 07207, Republic of Korea.; 6 Department of Biotechnology, College of Life Science and Biotechnology, Yonsei University, Seoul 03722, Republic of Korea.; 7 Prometabio Research Institute, Prometabio Co., Ltd. Hanam-si, Gyeonggi-do 12939, Republic of Korea.

**Keywords:** Synthetic lethality, NAMPT inhibitor, Epithelial-to-mesenchymal transition (EMT), Wallerian degeneration, Chemotherapy-induced peripheral neuropathy (CIPN)

## Abstract

**Background:** Exploiting synthetic lethality (SL) relationships between protein pairs has emerged as an important avenue for the development of anti-cancer drugs. Nicotinamide phosphoribosyltransferase (NAMPT) is the rate-limiting enzyme of the NAD+ salvage pathway, having an SL relationship with nicotinic acid phosphoribosyltransferase (NAPRT), the key enzyme in the NAD+ Preiss-Handler pathway. NAMPT inhibitor holds clinical potential not only as a promising cancer treatment but also as a means of protection against chemotherapy-induced-peripheral-neuropathy (CIPN). However, as NAD+ is essential for normal cells, the clinical use of NAMPT inhibitors is challenging. This study aimed to identify a novel NAMPT inhibitor with enhanced selective cytotoxicity against NAPRT-deficient cancer cells as well as prominent efficacy in alleviating CIPN.

**Methods:** We began by conducting drug derivatives screening in a panel of lung cancer cell lines to select an agent with the broadest therapeutic window between the NAPRT-negative and-positive cancer cell lines. Both in vitro and In vivo comparative analyses were conducted between A4276 and other NAMPT inhibitors to evaluate the NAPRT-negative cancer cell selectivity and the underlying distinct NAMPT inhibition mechanism of A4276. Patient-derived tumor transcriptomic data and protein levels in various cancer cell lines were analyzed to confirm the correlation between NAPRT depletion and epithelial-to-mesenchymal transition (EMT)-like features in various cancer types. Finally, the efficacy of A4276 for axonal protection and CIPN remedy was examined in vitro and in vivo.

**Results:** The biomarker-driven phenotypic screening led to a discovery of A4276 with prominent selectivity against NAPRT-negative cancer cells compared with NAPRT-positive cancer cells and normal cells. The cytotoxic effect of A4276 on NAPRT-negative cells is achieved through its direct binding to NAMPT, inhibiting its enzymatic function at an optimal and balanced level allowing NAPRT-positive cells to survive through NAPRT-dependent NAD+ synthesis. NAPRT deficiency serves as a biomarker for the response to A4276 as well as an indicator of EMT-subtype cancer in various tumor types. Notably, A4276 protects axons from Wallerian degeneration more effectively than other NAMPT inhibitors by decreasing NMN-to-NAD+ ratio.

**Conclusion:** This study demonstrates that A4276 selectively targets NAPRT-deficient EMT-subtype cancer cells and prevents chemotherapy-induced peripheral neuropathy, highlighting its potential as a promising anti-cancer agent for use in cancer monotherapy or combination therapy with conventional chemotherapeutics.

## Introduction

Nicotinamide adenine dinucleotide (NAD+) is an essential metabolite for cell survival; it not only serves as an oxidizing agent for energy production, fatty acid oxidization (FAO), and serine biosynthesis but also participates in numerous cellular processes as a substrate for various enzymes, such as SARM1 1, Sirtuins, and PARPs 2. The three pathways through which NAD+ can be supplied to cells include the salvage pathway from nicotinamide (NAM), the Preiss-Handler (PH) pathway from nicotinic acid (NA), and the *de novo* biosynthesis pathway from tryptophan. Cancer cells exhibiting accelerated proliferation as their acquired hallmark often show a high demand for NAD+ to support their survival 3. To fulfill their increased NAD+ demand, cancer cells augment NAD+ metabolic pathways. Nicotinamide phosphoribosyltransferase (NAMPT), the rate-limiting enzyme of the NAD+ salvage pathway is upregulated in various cancers, including gastric 4, colon 5, pancreatic 6, and breast cancers 7. Together with the growing potential of NAMPT as a therapeutic target for cancer treatment, numerous NAMPT inhibitors have been developed 8-11. Furthermore, research has expanded to involve novel approaches, such as dual inhibitors 12-15, antibody-drug conjugates 16, 17, and proteolysis-targeting chimeras (PROTACs) 18, 19.

**Figure 1:**
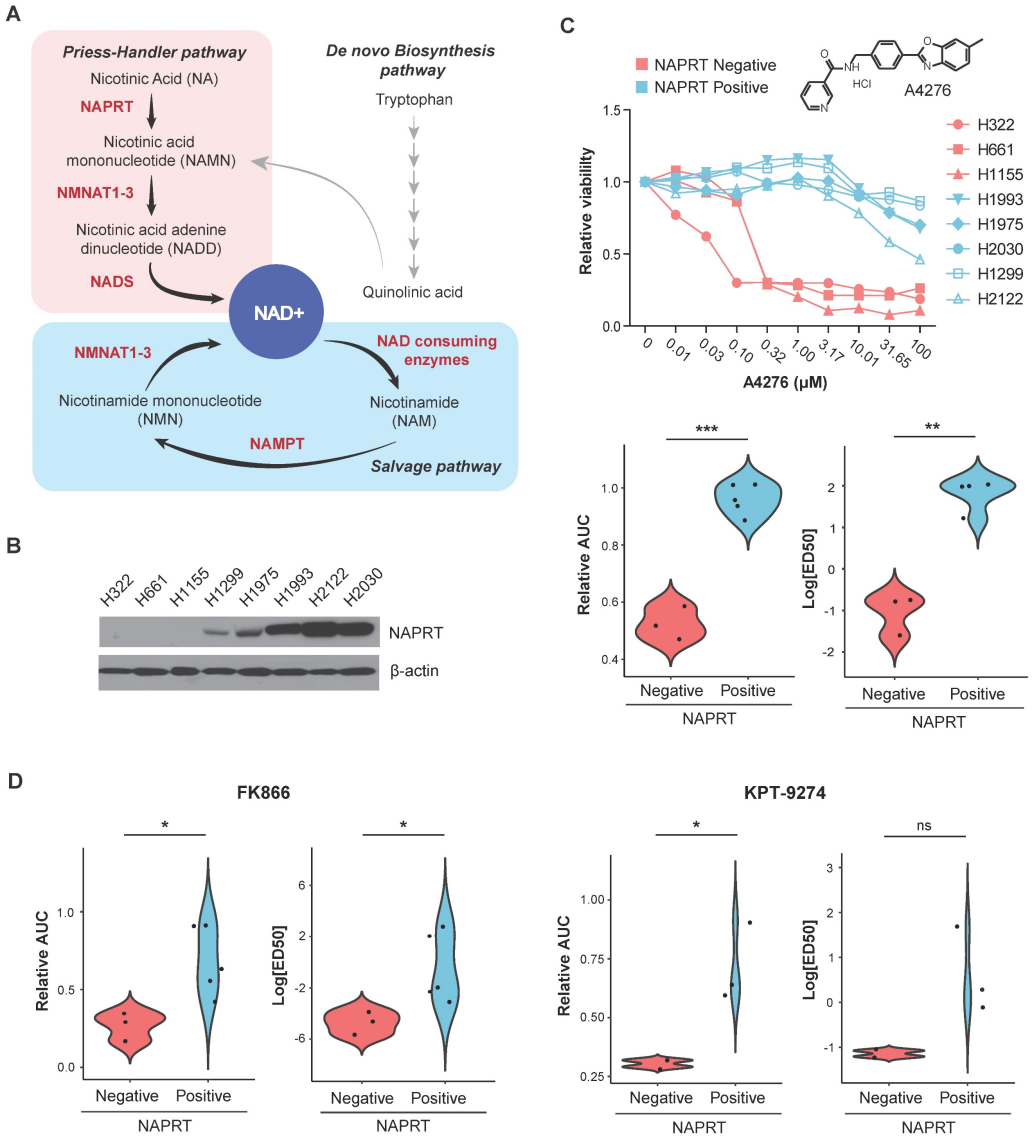
** Identification of A4276, a selective cytotoxic agent for NAPRT-deficient lung cancer cell lines. (A)** Schematic of NAD+ metabolism. Enzymes and metabolites related to NAD+ *de novo* biosynthesis pathway are not listed for simplicity. **(B)** Steady-state accumulation of NAPRT was assessed via immunoblot of whole-cell lysates from the indicated human lung cancer cell lines. Beta-actin was used as a loading control. **(C)** (Upper panel) The dose-response curves for eight different lung cancer cell lines after 72 hours of exposure to A4276 and chemical structure of A4276. The cell lines are categorized into two distinct groups based on their NAPRT status, NAPRT-positive (light blue) and NAPRT-negative (light red). (Lower panel) Violin plots of A4276 sensitivity for the two groups of lung cancer cell lines based on relative AUC and log_10_ ED50 values. **(D)** Violin plots representing the sensitivity of the lung cancer cell lines to FK866 (Left) and KPT-9274 (Right) based on relative AUC and log_10_ ED50 values. Dose-response curves are provided in Figure S1B-C. **(C, D)** *p < 0.05, **p < 0.01, ***p < 0.001, not significant (ns). Student's *t*-test was used for the comparison of drug sensitivity between the cell line groups.

Despite the growing progress in identifying NAMPT inhibitors, their therapeutic application is limited. Tumors can restore the NAD+ pool via alternative pathways, as shown in Figure 1A, thereby lowering the efficacy of NAMPT inhibitors. Additionally, severe NAD+ depletion can have lethal effects on normal cells, necessitating the candidate compounds with high selectivity against cancer cells with precise response biomarkers. In addition to NAMPT, nicotinic acid phosphoribosyltransferase (NAPRT), a critical enzyme in the PH pathway, contributes to the supply of NAD+. The complementary and independent action of NAPRT results in the poor efficacy of NAMPT inhibitors 20, 21. NAMPT and NAPRT, from the perspective of NAD+ production, can be regarded as having a synthetic lethality (SL) relationship, in which the perturbation of both genes leads to cell death, whereas no observable effects on cell viability occur when one member of the pair is inhibited 22. Therefore, in cancers with low-NAPRT expression, inhibition of NAMPT is a potential therapeutic approach.

The two hallmarks of cancer, EMT-like features and metabolic reprogramming, are not distinct cellular processes but rather interconnected phenomena 23. Extensive research has established a strong link between alterations in the NAD+ synthetic pathway and development of EMT-like features as well as chemotherapy resistance in various cancer types 5, 24. Furthermore, our previous study revealed NAPRT downregulation as a molecular feature of EMT-subtype gastric cancer cells 21. These findings support the intricate association between EMT, modulation of the NAD+ synthetic pathway, and therapy resistance. Consequently, it becomes imperative to identify effective NAMPT inhibitors that selectively target NAPRT-deficient cancers, both as monotherapy and in combination with standard chemotherapy, to overcome the challenges posed by potentially malignant tumors.

Neuronal NAD+ levels are closely linked to axonal survival and death 25. A prolonged decline in NAD+ concentration in the axon activates an irreversible local self-destruction program, which leads to axon degeneration and, eventually, neuronal cell death. This axon destruction pathway, which is conserved from Drosophila to humans, is dependent on SARM1, a novel NAD+-consuming enzyme 26. SARM1 activity is maintained in healthy axons, where NAD+ levels are continuously replenished by the salvage pathway, which involves the conversion of nicotinamide mononucleotide (NMN) to NAD+. Mechanical injuries (such as nerve compression) and/or chemical insults (such as chemotherapy) inhibit axonal protein transport, resulting in the depletion of labile nicotinamide mononucleotide adenylyltransferase 2 (NMNAT2) protein 27. This leads to a decrease in NAD+ and an increase in NMN, thereby raising the NMN-to-NAD+ ratio, which triggers activation of SARM1, and leads to catastrophic consumption of NAD+. This axon destruction pathway is believed to be a mechanism shared by many neurodegenerative diseases, including chemotherapy-induced peripheral neuropathy (CIPN), a common but detrimental side effect of chemotherapies such as taxanes and platinums. Therefore, preventing NAD+ loss (by inhibiting SARM1) or NMN gain (by partially inhibiting NAMPT) delays axon degeneration and is hailed as a new therapeutic approach for treating, delaying, or preventing neurodegeneration. These findings signify the therapeutic potential of NAMPT inhibitors not only as cancer-targeting single agents, but also as a promising strategy for unmet medical needs in oncology by preventing detrimental chemotherapy-induced adverse effects when applied in combination with commonly used chemotherapies.

In this study, we identified a novel, orally bioavailable NAMPT inhibitor, A4276, using phenotypic screening to emphasize its selective cytotoxicity against NAPRT-negative cancer cells. We confirmed that NAPRT deficiency can serve as an indicator of EMT-like cancers across various tumor types, underscoring the clinical significance of our compound. Furthermore, considering the broad role of the NAD+ biosynthetic pathway in governing diverse cellular processes, we extended the potential applications of our compound to CIPN, a condition for which manipulating the NMN-to-NAD+ ratio has reported relevance as a potential treatment. Overall, our study identified a novel compound, A4276, with great potential as a specific treatment for NAPRT-deficient EMT-like cancers. Additionally, A4276 shows promise as a protective agent against axonal death, potentially alleviating adverse effects when used in combination with chemotherapy.

## Results

### Identification of A4276, a selective cytotoxic agent for NAPRT-deficient lung cancer cell lines

As NAMPT and NAPRT are the rate-limiting enzymes of the two NAD+ biosynthetic pathways (Figure 1A), neoplastic depletion of NAPRT can serve as a biomarker of NAMPT inhibitor-sensitive cancer. Starting with SW008135, a small-molecule compound whose cytotoxicity is negatively correlated with *NAPRT* expression as a single biomarker 28, we screened SW008135 derivatives in a panel of lung cancer cell lines with varying NAPRT expression to improve potency and selectivity. The panel included eight human lung cancer cell lines; three NAPRT-negative and five NAPRT-positive (Figure 1B). Based on the wider margin between lethal doses in NAPRT-negative versus NAPRT-positive cell lines compared to the hit compound SW008135 (Figure S1A) and other derivatives (data not shown), A4276 was selected for further investigation. The lower area under the curve (AUC) and 50% effective dose (ED50) values in NAPRT-negative cell lines demonstrated that A4276 selectively killed NAPRT-deficient lung cancer cell lines (Figure 1C). Notably, the selective margin of A4276 was wider than that of the other well-known NAMPT inhibitors, FK866 and KPT-9274 (Figure 1C-D, Figure S1B-C). In summary, we identified A4276 as a novel compound with high selectivity for NAPRT-deficient lung cancer cell lines.

### A4276 inhibits the enzymatic function of NAMPT

**Figure 2:**
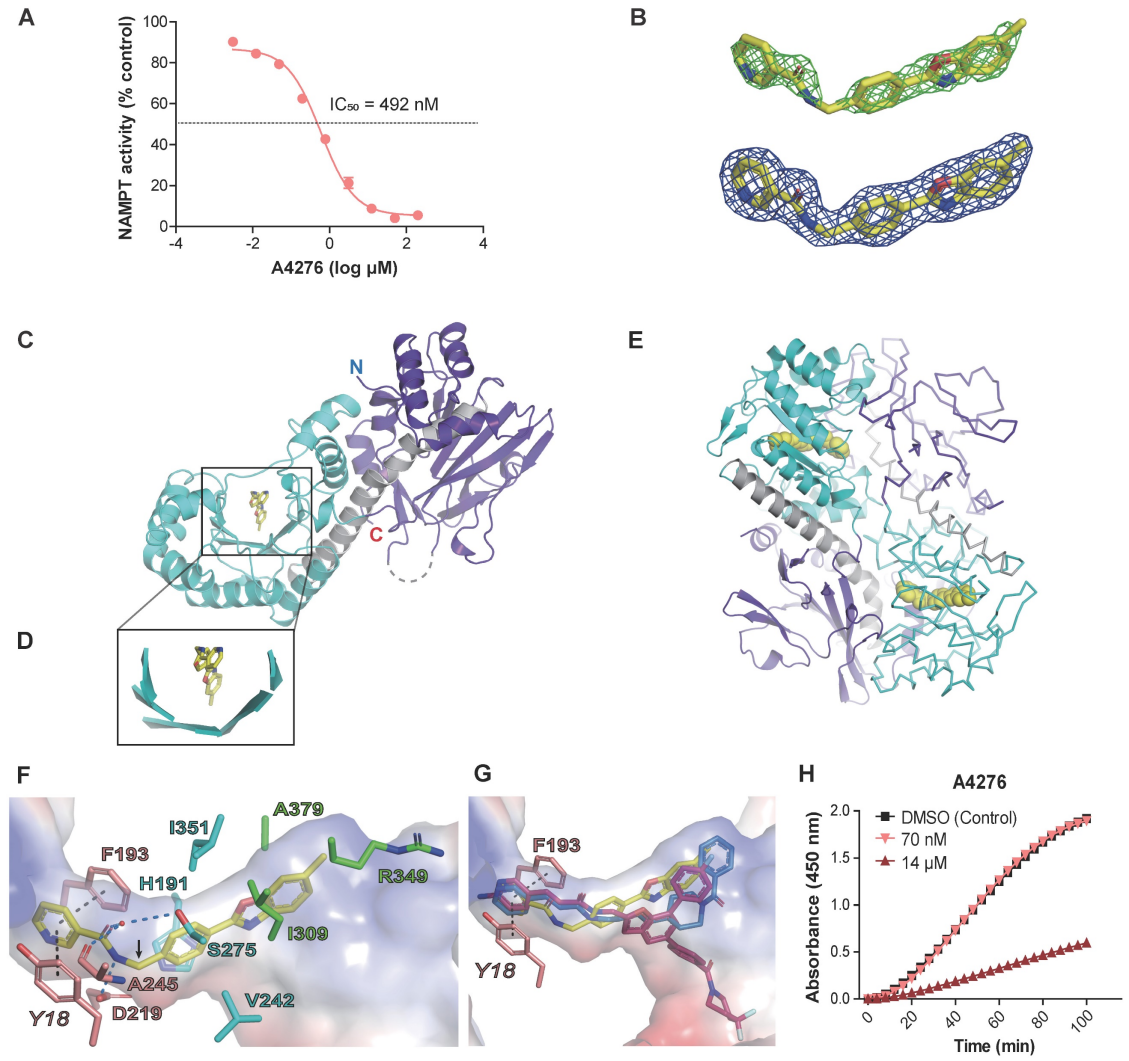
** A4276 inhibits the enzymatic function of NAMPT. (A)** The assays for NAMPT enzyme activity confirms the concentration-dependent inhibition of NAD+ synthesis by A4276.** (B)** Electron density maps show A4276 (yellow sticks) bound to the active site of NAMPT. The simulated annealing *F*_o_-*F*_c_ electron-density maps (green) contoured at 3 σ and the final maximum-likelihood weighted 2*F*_o_-*F*_c_ electron density maps (blue) contoured at 1 σ. **(C)** Monomeric NAMPT structure with the first and second domains and helix α5 are colored in purple, cyan and gray, respectively. A4276 is indicated by a yellow stick. 'N' and 'C' represent the N-terminal and C-terminal ends, respectively. **(D)** Close-up view of the square region in (C). **(E)** Dimeric NAMPT structure with molecule A shown in the ribbon and molecule B shown in cartoons for clarity. A4276 is shown as a yellow sphere at the active site. **(F)** Binding mode of A4276 to subsite-I (pink sticks), subsite-II (cyan sticks), and subsite-III (green sticks). *Tyr18*, Phe193, Asp219, and Ala245 constitute subsite-I. His191, Val242, Ser275, and Ile351 constitute subsite-II. Ile309, Arg349 and Ala379 constitute subsite-III. The black dotted lines indicate π-π stacking interactions, and blue dotted lines indicate hydrogen bonds. The kink at the C10 atom of A4276 is indicated by an arrow. The transparent surface represents the electrostatic potential of the active site. **(G)** Superimposed structures of A4276 (yellow; PDB entry 8ivu), FK866 (blue; PDB entry 2gvj), and KPT-9274 (magenta; PDB entry 5nsd). **(H)** Time courses of NAMPT reactivation. Time-dependent spectral changes at 450 nm under 1.5% of DMSO (black squares), 14 µM of A4276 (red triangles), and 70 nM of A4276 (light red, inverted triangles) are presented.

We hypothesized that A4276 has high selectivity for NAPRT-deficient cancer cells due to it inhibiting NAMPT enzymatic activity, as these cancer cells are likely dependent on NAMPT for NAD+ supply. To test this hypothesis, we treated the purified NAMPT with various concentrations of A4276 and measured its activity (Figure 2A). We observed a significant decrease in NAD+ production by NAMPT in a concentration-dependent manner for A4276 (IC50 of 492 nM), surpassing the inhibitory potency of the hit compound (IC_50_ of 2.27 μM, Figure 2A and Figure S2).

To determine whether A4276 inhibits NAMPT through direct binding, and to elucidate its mechanism of action, we solved the crystal structure of the NAMPT-A4276 complex which showed clear electron densities for A4276 (Figure 2B). NAMPT exists as a homodimer in solution 29, 30. The asymmetric unit of the crystal structure contains two dimers of the complex, with dimer-1 composed of molecules A and B, and dimer-2 composed of molecules C and D. The crystal structures of dimer-1 and dimer-2 were superimposed with a root-mean-square deviation of 0.204 Å for all equivalent Cα atoms. Since the two dimers have virtually identical structures, we selected dimer-1 for structural description. Monomeric NAMPT has a modular structure consisting of two domains. The first domain, which consists of two segments from the N- and C-terminal regions (residues 8-141 and 391-483), has a six-stranded antiparallel β-sheet with five α-helices on one face. The other face of this β-sheet is covered by a two-stranded antiparallel β-sheet and an α-helix (Figure 2C). The second domain (residues 181-390) contains a U-shaped central β-sheet formed by five parallel β-strands surrounded by nine α-helices (Figure 2C-D). The two domains are connected by a long helix α5 (residues 142-180) (Figure 2C). Two monomers are arranged to form a dimer in a head-to-tail manner, where the first domain of one monomer is assembled with the second domain of the other monomer to form the active site, indicating the presence of two active sites in the dimer (Figure 2E). For clarity, residues in monomer A are denoted in normal font, whereas those in monomer B are denoted in italics.

A4276 nestles in the U-shaped central β-sheet of the second domain (Figure 2D), adopting a V-shaped conformation with a kink at the C10 atom (Figure 2F). The structure of A4276 comprises three parts: nicotinamide, benzene, and 6-methylbenzoxazole groups. The active site of NAMPT can be divided into subsites-I, -II, and -III, which accommodate nicotinamide, benzene, and 6-methylbenzoxazole, respectively (Figure 2F). Through extensive π-π stacking interactions, the pyridine ring of nicotinamide is trapped between *Tyr18* and Phe193 of subsite-I, where the substrate nicotinamide ring binds (Figure 2F). The amide group of A4276 makes polar interactions with Asp219 and a water molecule in subsite-I; the -NH group is hydrogen-bonded to the side-chain carboxylate of Asp219, and the carbonyl oxygen is connected to a water molecule interacting with Ser275 and Ala245 (Figure 2F). The benzene ring of A4276 is nestled in the hydrophobic subsite-II lined by His191, Val242, Ile351, and the Cβ atom of Ser275 (Figure 2F).

Notably, the nicotinamide and benzene rings are buried in the narrow tunnel constituted by subsites-I and -II (Figure 2F), which allows tight contacts with the active site. In contrast, only one side of 6-methylbenzoxazole interacts with Ala379, Ile309, and the hydrophobic part of the side chain of Arg349 in subsite-III, while the other side is exposed to the solvent (Figure 2F). The distinct exposure states of the three rings in A4276 are reflected in their solvent accessible surface areas; 9.306 Å^2^, 0.227 Å^2^, and 39.720 Å^2^ for nicotinamide, benzene, and 6-methylbenzoxazole, respectively. In addition, the higher thermal *B*-factor value of 6-methylbenzoxazole indicates its relatively weak binding with *B*-factors of 36.07, 32.44, and 38.66 Å^2^ for nicotinamide, benzene, and 6-methylbenzoxazole, respectively. Subsite-III is open to solvent, in contrast to the tunnel-like subsites-I and -II, which makes it suitable for the binding of bulky groups. When comparing A4276 with FK866 and KPT-9274, the nicotinamide group of A4276 and corresponding moieties of the other two compounds are similarly positioned, engaging in stacking interactions with *Tyr18* and Phe193 (Figure 2F-G). However, the remaining parts of the three inhibitors have different poses (Figure 2G), indicating distinct interactions with the active site residues of NAMPT. Overall, the crystal structure revealed that A4276 binds to the active site of NAMPT exclusively through non-covalent interactions. Supporting these data, NAMPT inhibited by the excess A4276 displayed a time-dependent gradual reactivation (Figure 2H). Furthermore, the activity of the inhibited NAMPT rapidly recovered to the level of enzyme activity under DMSO control after a 200-fold dilution of A4276 (Figure 2H). Collectively, the structural data and NAMPT enzyme activity assays support the notion that A4276 confers a growth-inhibitory effect on cancer cells with decreased NAPRT levels by directly binding to and reversibly inhibiting the activity of NAMPT.

### NAPRT-depletion represents an EMT-like feature and renders various types of tumors sensitive to A4276

**Figure 3:**
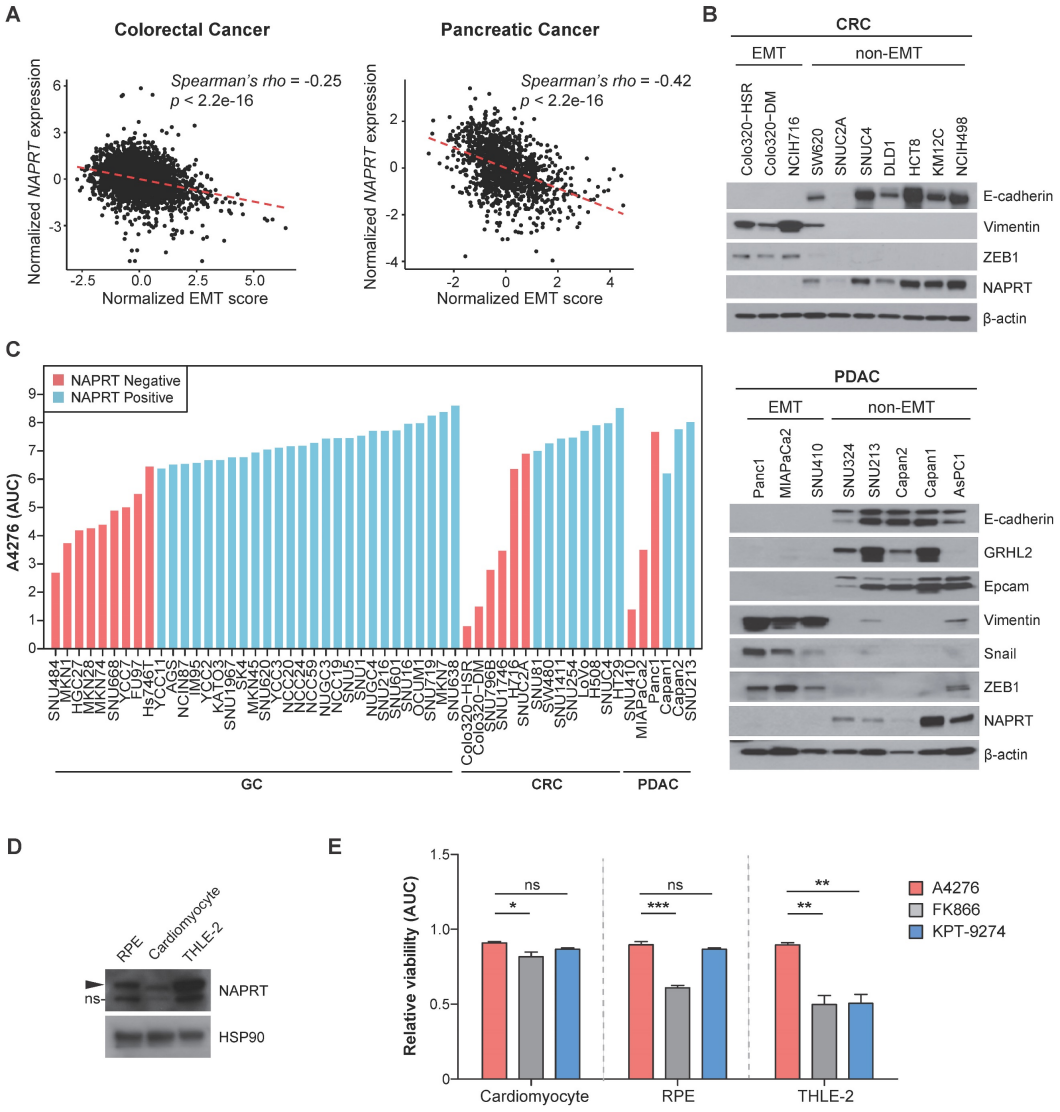
** NAPRT-depletion represents an EMT-like feature and renders various types of tumors sensitive to A4276. (A)** Graphs showing a correlation between the normalized EMT score and *NAPRT* expression in colorectal (left) and pancreatic (right) tumor tissues. EMT score and *NAPRT* expression level of each tumor type were calculated based on gene expression data collected from multiple public cohorts. See methods for details. Statistical significance of the correlation was assessed using Spearman's rank correlation test. **(B)** Steady-state accumulation of the indicated proteins was assessed via immunoblot of whole cell lysates from the indicated colorectal (upper) and pancreatic (lower) cancer cell lines. Beta-actin was used as a loading control. **(C)** The area under the curve (AUC) values of the indicated cancer cell lines were calculated based on dose-response curves after 72 h of exposure to a various concentration of A4276. Each cancer cell line was classified into NAPRT-negative (light red) or NAPRT-positive group (light blue). GC; gastric cancer, CRC; colorectal cancer, PDAC; pancreatic ductal adenocarcinoma. See methods for details. **(D)** Steady-state accumulation of NAPRT in the indicated normal cells was assessed via immunoblot of whole cell lysates. HSP90 was used as a loading control. The band labeled with ns indicates a non-specific band. **(E)** Comparison of the normal cell toxicities elicited by the three different NAMPT inhibitors. The area under the curve (AUC) values were calculated from the dose-response curves obtained after treating each cell line with indicated compounds for 72 h. Dose-response curves are provided in Figure S4. *p < 0.05, **p < 0.01, ***p < 0.001, not significant (ns). Ordinary one-way ANOVA, followed by Tukey's multiple comparison tests, was used for the comparison of the normal toxicities of NAMPT inhibitors.

In light of our previous report demonstrating a strong correlation between NAPRT downregulation and the EMT status of cancer cells in gastric cancer (GC) 21, we investigated whether this trend is conserved in other cancer types. We analyzed multi-cohort transcriptomics datasets of colorectal cancer (CRC) and pancreatic ductal adenocarinoma (PDAC) to determine the relationship between the extent of EMT-like features, estimated here as the EMT score, and *NAPRT* expression in the tumor tissues. We observed a significant inverse correlation between the EMT score and *NAPRT* abundance in both CRC and PDAC tissues (Figure 3A). To further investigate this relationship at the molecular level, we conducted immunoblotting using various CRC and PDAC cell lines (Figure 3B). As expected, cell lines with depleted NAPRT expression exhibited EMT-like molecular features, as evidenced by the expression patterns of EMT marker proteins, such as Vimentin, ZEB1, and Snail, and the lack of expression of epithelial cell markers, such as E-cadherin, GRHL2, and EpCAM (Figure 3B). These results confirmed the negative correlation between NAPRT expression and the extent of EMT-like features at both the tissue and cell line levels, and led us to conclude that NAPRT deficiency can serve as a biomarker for EMT-subtype cancer in a variety of tumor types, including but not limited to GC, CRC, and PDAC.

These data imply the clinical potential of A4276 for the treatment for various tumor types of malignant EMT-like cancer characterized by downregulated NAPRT. To further demonstrate this potential, we evaluated the responses of diverse GC, CRC, and PDAC cell lines to A4276 treatment. Regardless of the cancer type, NAPRT-deficient cancer cell lines were more susceptible to A4276 than the NAPRT-positive cancer cell lines (Figure 3C). The inverse correlation between the response to A4276 and NAPRT level was further validated through transcriptomic analysis of the cancer cell lines used (Figure 1B-D, Figure 3C), for which expression data were available in the DepMap dataset (Figure S3A). Additionally, we addressed the controversy surrounding the correlation between NAMPT expression levels and responses to NAMPT inhibitors 10, 31, 32. In the same cancer cell lines, we did not observe a substantial correlation between the response to A4276 and NAMPT level (Figure S3B). The capacity of sparing NAPRT-positive cell lines is important, considering that the major issue with the current NAMPT inhibitors in clinical trials is their toxicity to normal cells. Therefore, we evaluated the response of normal cells against A4276. The expression of NAPRT in three normal cell lines, cardiomyocytes, retinal pigment epithelium (RPE) cells, and human adult hepatocyte cells (THLE-2), was confirmed by immunoblotting (Figure 3D), and none of the normal cells were sensitive to A4276 treatment (Figure 3E and Figure S4). In contrast, at least one type of normal cell was affected by other NAMPT inhibitors (Figure 3E and Figure S4). In conclusion, the results strongly suggest that A4276 is an NAMPT inhibitor that exhibits remarkable selectivity against NAPRT-deficient EMT-like cancer cell lines across various cancer types while effectively sparing NAPRT-positive cells, particularly, normal cells.

### Intact NAPRT/NAD+ flux confers robust resistance of NAPRT-positive cells to A4276

The specific cytotoxicity of A4276 sparing NAPRT-positive cell lines in multiple cancer types and normal cells (Figure 1C, Figure 3C, and 3E) led us to propose three hypotheses regarding the nature of NAMPT inhibitors. First, the cytotoxicity of the other NAMPT inhibitors observed in certain NAPRT-positive cell lines can be attributed to off-target toxicity. Second, A4276 may have secondary targets whose regulation by A4276 may protect NAPRT-positive cells from the detrimental effects of NAD+ depletion. Finally, the degree of NAMPT inhibition may vary among the inhibitors, and that induced by A4276 might fall within a "Goldilocks zone." This implies that the potency of A4276 as a NAMPT inhibitor is sufficient to deplete NAD+ below the necessary threshold required for cell survival in NAPRT-negative cells, yet, not excessive to surpass the capacity of bypass NAD+ supply from NA in the presence of NAPRT. To test these hypotheses, we compared the cellular and enzymatic effects of A4276 and other NAMPT inhibitors. Among the NAMPT inhibitors used in our study, we selected FK866 instead of KPT-9274, which has a secondary target, PAK4, that could introduce complexities in interpreting the results. Furthermore, the inhibitory kinetics of FK866 on NAMPT have been relatively well-documented compared to KPT-9274. FK866 is a tight-binding, competitive inhibitor of NAMPT 29; therefore, we compared A4276 with FK866 to gain insights into the mechanism underlying A4276's superior selectivity against NAPRT-negative cancer cell lines.

**Figure 4:**
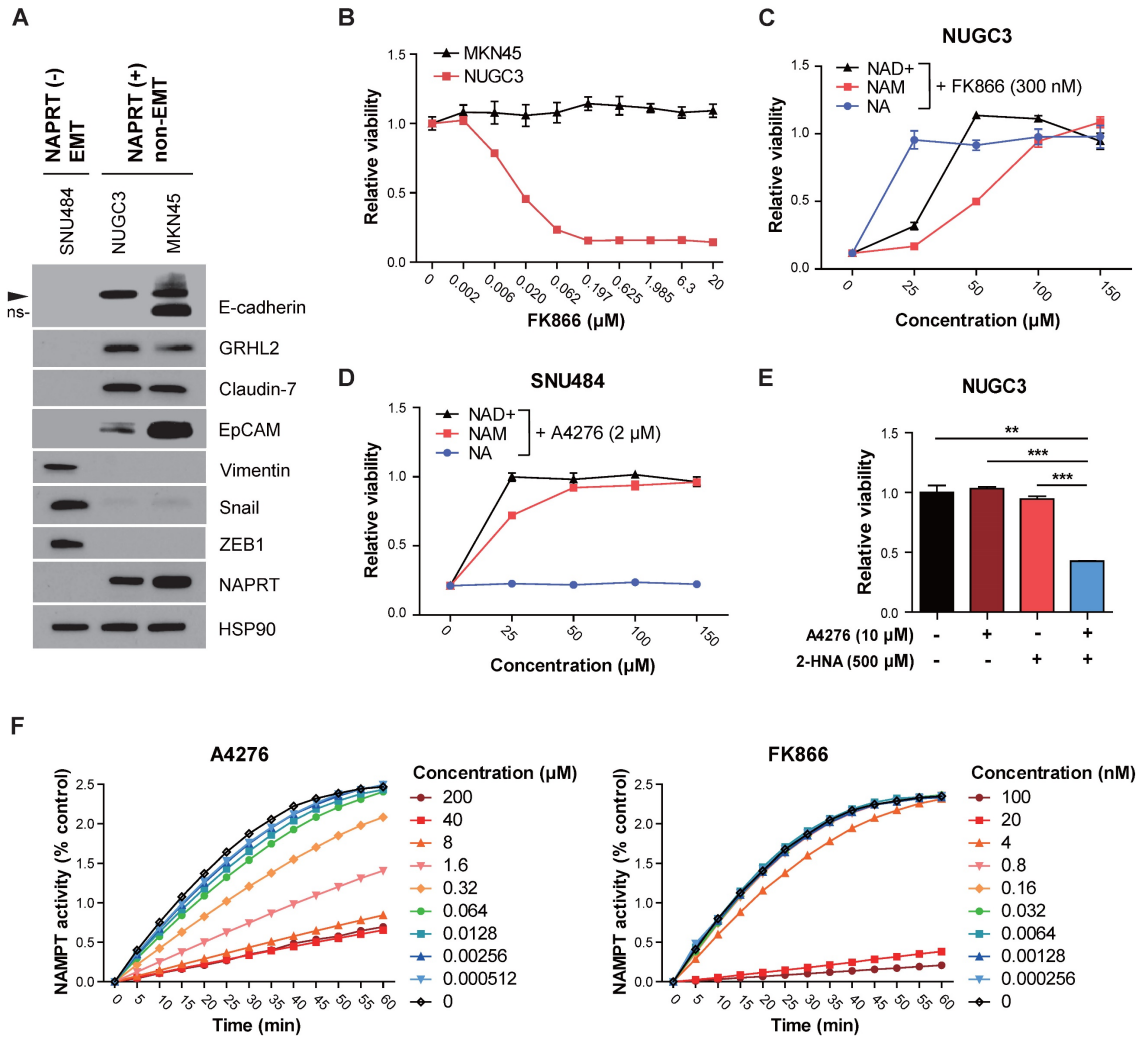
** Intact NAPRT/NAD+ flux confers robust resistance of NAPRT-positive cells to A4276. (A)** Steady-state accumulation of the indicated proteins was assessed via immunoblot of whole-cell lysates from the indicated cancer cell lines. SNU484, NUGC3, and MKN45 represent three distinct classes of cell lines based on their responses to A4276 and FK866. HSP90 was used as a loading control. The band labeled with ns indicates a non-specific band. **(B)** Dose-response curves for indicated NAPRT-positive non-EMT cancer cell lines after 72 h of exposure to a various concentration of FK866. The relative viability of indicated cell lines were assessed after 72 h of treatment with **(C)** FK866 or **(D)** A4276 in combination with NAD+, nicotinamide (NAM) or nicotinic acid (NA) at the indicated concentrations. **(E)** The relative viability of NUGC3 cell line was assessed after treatment with the indicated compounds. Statistical significance was determined using Student's *t*-test, with **p < 0.01 and ***p < 0.001, indicating significance.** (F)** The time course activity of NAMPT inhibited by the indicated compounds. The activity of NAMPT was monitored every 5 minutes over a 60-minute period after 60 minutes of incubation with each compound at the presented concentrations. **(B-E)** Error bars indicate the mean ± SD (n = 3).

**Figure 5:**
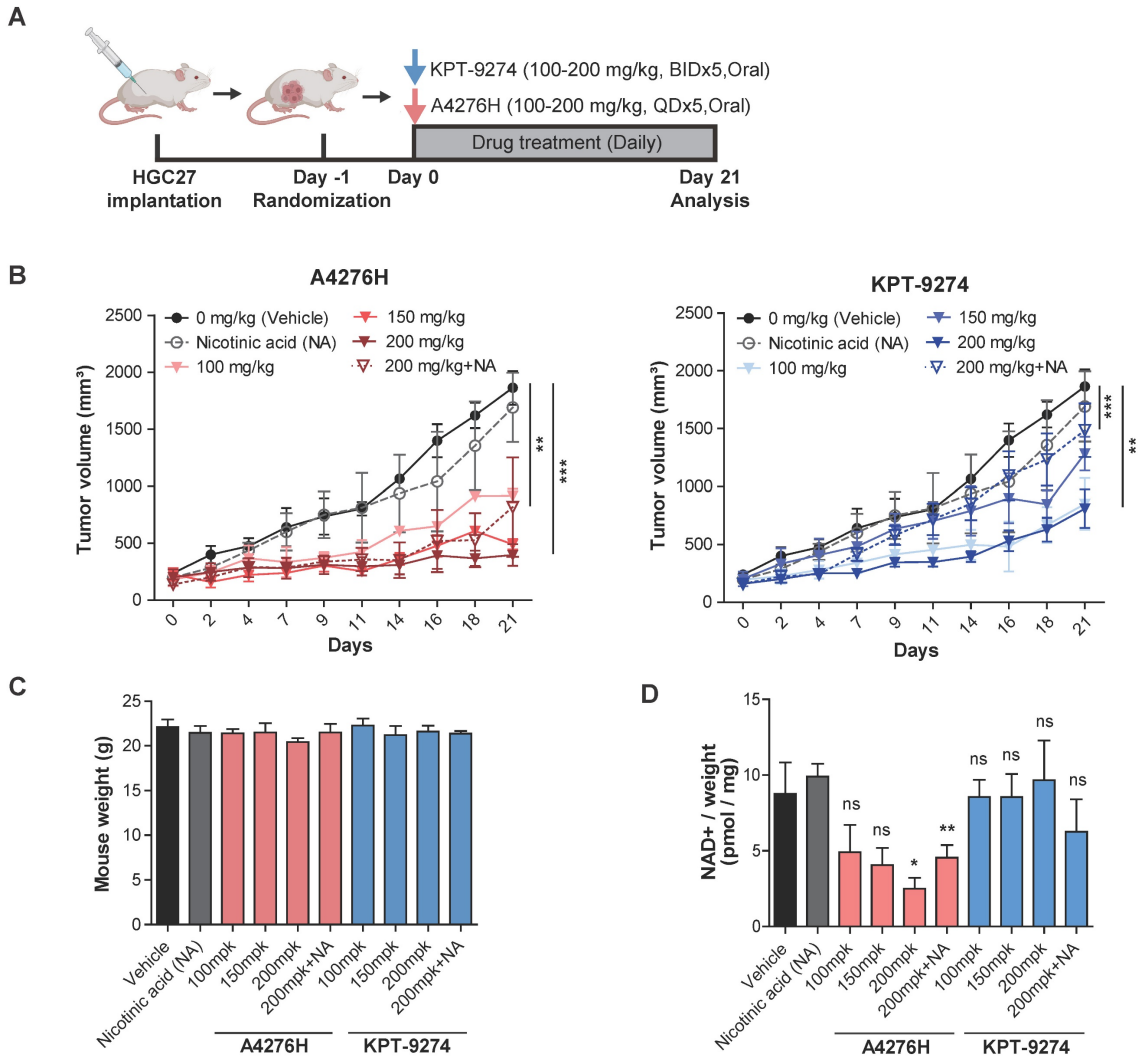
** A4276 potently inhibits the growth of NAPRT-negative EMT-subtype cancer *in vivo.* (A)** Experimental scheme comparing the *in vivo* efficacy of A4276H and KPT-9274 in HGC27 xenografts. BID: twice a day, QD: once a day.** (B)** *In vivo* anti-tumorigenic efficacy of the indicated compounds in HGC27 xenografts. Each group of mice was treated with the agent or vehicle with or without 100 mg/kg of NA, and tumor volumes were measured on the indicated days. Error bars indicate the mean ± SEM (n= 6-7 per group). **p < 0.01 and ***p < 0.001. Statistical significance was determined using two-way ANOVA, followed by Tukey's multiple comparison tests. **(C)** Graphs showing mean mouse weight for each group measured at the endpoint of the experiment. **(D)** The Total NAD+ levels measured in the tumor mass relative to the weight of the mice in each group at the endpoint. Error bars indicate mean ± SEM (n= 6-7 per group). *p < 0.05, **p < 0.01, not significant (ns). Student's *t*-test was conducted to compare the groups treated with the test compound with the vehicle-treated group, except for the comparison between the 100 mg/kg A4276H and vehicle groups, where the Wilcoxon rank-sum test was conducted. Groups treated with the NAMPT inhibitor were compared with the vehicle-treated group, whereas the groups supplemented with NA were compared with the NA-treated group.

To test the first hypothesis, we examined whether certain NAPRT-positive cancer cell lines sensitive to FK866 are subjected to NAM-competitive NAMPT inhibition and NAD+ depletion by FK866. We observed that NUGC3 cells harbor NAPRT-positive and non-EMT features but were resistant only to A4276 (Figure 3C, 4A-B). The response against FK866 in NUGC3 cells was completely blocked by the co-administration of NAD+ and NAM, indicating that FK866 induced cell death primarily by targeting the NAD+ salvage pathway through NAM-competitive inhibition (Figure 4C). Furthermore, the observed inhibition of FK866 toxicity by NA suggested basal capacity of incorporating NA to NAD+ in this cell line is weak, and enhancing the efficacy of the NAPRT-dependent NAD+ supply can protect it from FK866-induced cell death (Figure 4C). Such tendencies were further confirmed in two additional cell lines, KM12C and DLD-1, which are NAPRT-positive and non-EMT but resistant only to A4276 (Figure 3B, and Figure 5SA-B).

After excluding the first hypothesis, we proceeded to assess the potential involvement of a secondary target in the A4276's selectivity. To verify whether the A4276's toxicity depends on the perturbation of the NAD+ synthetic pathway, we supplemented NAD+, NAM, and NA with A4276 in NAPRT-negative EMT-cancer cell lines exhibiting sensitivity to A4276, SNU484 (Figure 3C and 4A), and additionally COLO320-HSR (Figure 3B-C). Both NAD+ and NAM effectively prevented cell death induced by A4276, whereas NA did not, suggesting that the cytotoxicity of A4276 is also mediated by NAM-competitive perturbation of the salvage pathway and NAD+ depletion (Figure 4D and Figure S5C). Moreover, treating NUGC3 cells with the NAPRT inhibitor 2-HNA in combination with A4276 resulted in cytotoxicity (Figure 4E), demonstrating that the Preiss-Handler pathway was the major contributor in protecting this NAPRT-positive cell line against A4276. In addition, we conducted a comparative assessment of NAD+ concentrations with NUGC3 and SNU484 cells after treating FK866 and A4276 at concentrations where 72-hour treatment of each compound led to apparent and comparable cell death in the responsive cell lines (Figure S5D-E). As expected, a 24-hour exposure to both compounds substantially reduced total NAD levels in a similar manner in NAPRT-negative SNU484 cells (Figure S5E). However, the significance of the interaction between cell type and treatment (p < 0.001, two-way ANOVA) revealed that the NAD reductive effect of each drug varied across cell lines, likely attributed to a more pronounced reduction in NAD levels by FK866 compared to A4276 in NUGC3 cells (Figure S5E). Taken altogether, we propose that A4276 inhibits NAMPT at an optimal and balanced level allowing NAPRT-positive cells to survive through NAPRT-dependent NAD+ synthesis, unlike FK866, which causes excessive NAMPT disruption, thereby leading to the undesired death across several NAPRT-positive cancer cell lines with less efficient NAPRT/NAD+ flux.

Additionally, we conducted a comparative analysis of A4276 and FK866's mode of NAMPT inhibition. We treated purified NAMPT with different concentrations of each of the inhibitors, and monitored its enzymatic activity over 60 min (Figure 4F). Notably, the enzyme activity recovery rate of NAMPT after maximal inhibition by FK866 was extremely slow compared to A4276, indicating its tight-binding nature 29, and differences in the modes of NAMPT inhibition between the two drugs. Altogether, these results suggest that A4276 possess a distinct mode of enzyme inhibition compared to FK866, which may lead to an optimal range of NAMPT inhibition instead of strict perturbation like FK866, thus preventing NAPRT-positive cells from lethal NAD+ depletion.

### A4276 potently inhibits the growth of NAPRT-negative EMT-subtype cancer *in vivo*

Based on the promising *in vitro* NAPRT-deficient and EMT-like cell selective potency of A4276, we investigated the *in vivo* efficacy of A4276. For this assessment, we used the HCl salt form of A4276 (A4276H) to enhance its water solubility. Furthermore, we explored the potential benefits of co-administration with nicotinic acid (NA) to extend the therapeutic window of the agent. Previous reports have suggested that NA supplementation can prevent normal cell toxicity associated with NAMPT inhibition by restoring a sufficient NAD+ in normal cells while rarely affecting NAPRT-deficient tumor cells 15, 33. Before exploring the *in vivo* therapeutic potential of A4276, we assessed its cardiac toxicity and pharmacokinetics (PK). To determine if A4276 is associated with cardiac dysfunction, we conducted an *in vitro* human ether-à-go-go-related gene (hERG) cardiac potassium channel inhibition assay, with testing concentrations ranging from 0.001 μM to 100 μM. The positive control, E-4031, inhibited hERG currents by 92.8% (± 6.11%) at 10 μM, while A4276H inhibited hERG currents by only 14.6% (± 1.84%) at 10 μM (Table S1). Indeed, all tested concentrations of A4276H resulted in hERG current inhibition of less than 50% (Table S1). Subsequently, we determined the PK parameters following oral administration of A4276. The maximum drug concentration (C_max_) and total drug exposure (AUC_last_) increased in a dose-proportional manner. Interestingly, the AUC_last_ at a dose of 160 mg/kg and when co-administered with NA were comparable in both plasma and tumor tissues (Figure S6). Furthermore, the PK analysis revealed a half-life indicative of once or twice daily oral dosing of A4276, as the time to reach maximum plasma concentration (T_max_) ranged from two to eight hours, followed by a gradual decline over 24 h in all groups (Figure S6).

After confirming the absence of hERG-mediated cardiac toxicity and assessing the PK properties, we investigated the *in vivo* efficacy of A4276. To validate its potential translational applications, we conducted a head-to-head comparison with KPT-9274, the only NAMPT inhibitor currently being assessed in phase I clinical trials for treating advanced solid tumors or hematologic malignancies, rather than FK866, the clinical trials of which have been terminated due to dose-limiting toxicities. To test the efficacy of A4276H and KPT-9274, we treated mice bearing HGC27 tumors with both NAMPT inhibitors when the tumors reached a volume of approximately 150 mm^3^ (Figure 5A). The dosing schedule of KPT-9274 was based on a previously reported *in vivo* efficacy study of KPT-9274 in a renal cell carcinoma xenograft model 15. Both agents induced tumor regression without significant weight loss after 21 days of treatment at doses of 100, 150, 200, and 200 mg/kg co-administered with 100 mg/kg of NA (Figure 5B-C). Notably, despite KPT-9274 being administered twice as frequently as A4276, A4276 showed superior anti-tumor effects in most cases, as demonstrated by higher tumor-growth inhibition (Figure 5B). It was also of interest to determine whether the drugs could retain their anti-cancer effects even when delivered in combination with NA. When comparing the groups exposed to the agents at a dose of 200 mg/kg and those supplemented with NA, the greater inhibitory efficacy of A4276 was reinforced; at a dose of 200 mg/kg, A4276H inhibited tumor growth by 83.75%, whereas KPT-9274 inhibited it by 60.10% (two-way ANOVA, *p* < 0.0001, *p* = 0.00269, respectively). When combined with NA, A4276H suppressed tumor growth by 58.27%, whereas KPT-9274 only inhibited it by 18.45% (two-way ANOVA, *p* = 0.00906, *p* < 0.0001, respectively) (Figure 5B).

To confirm the target inhibitory activity of A4276 and KPT-9274, we measured the relative NAD+ levels in tumor tissues. As expected, A4276H treatment led to a significant dose-dependent reduction in NAD+ levels compared with KPT-9274 treatment (Figure 5D), indicating that A4276H more effectively inhibited NAMPT *in vivo* than KPT-9274. These results support the superior anti-tumor efficacy of A4276 over KPT-9274, regardless of NA supplementation, and suggest that A4276 has significant potential as a safe and effective NAMPT inhibitor for the treatment of NAPRT-deficient EMT cancers.

After identifying A4276 as a biomarker-selective single-agent anticancer therapy, we investigated its potential to synergistically target cancer cells in combination with standard chemotherapies. Conventional chemotherapeutic regimens have limited efficacy due to severe off-tumor toxicity and the development of resistance, which has led to their combination with targeted therapeutics as a promising solution. Therefore, we evaluated the anti-cancer cytotoxicity of A4276 in combination with the commonly used chemotherapeutic agent, paclitaxel (Figure S7). The two compounds were co-treated at various concentrations to the cancer cell employed in our preclinical study, HGC27 and additionally, COLO320-HSR (Figure S7A). Using the Chou-Talalay method 34, combination index (CI) score was computed, revealing CI values within the synergistic range (CI < 1) (Figure S7B). The synergistic efficacy of A4276 and paclitaxel was consistently observed across a wide range of combination concentrations in both cancer cell lines, providing a strong rationale for combining chemotherapy with A4276 in the treatment of advanced solid malignancies.

### A4276 delays Wallerian degeneration

**Figure 6:**
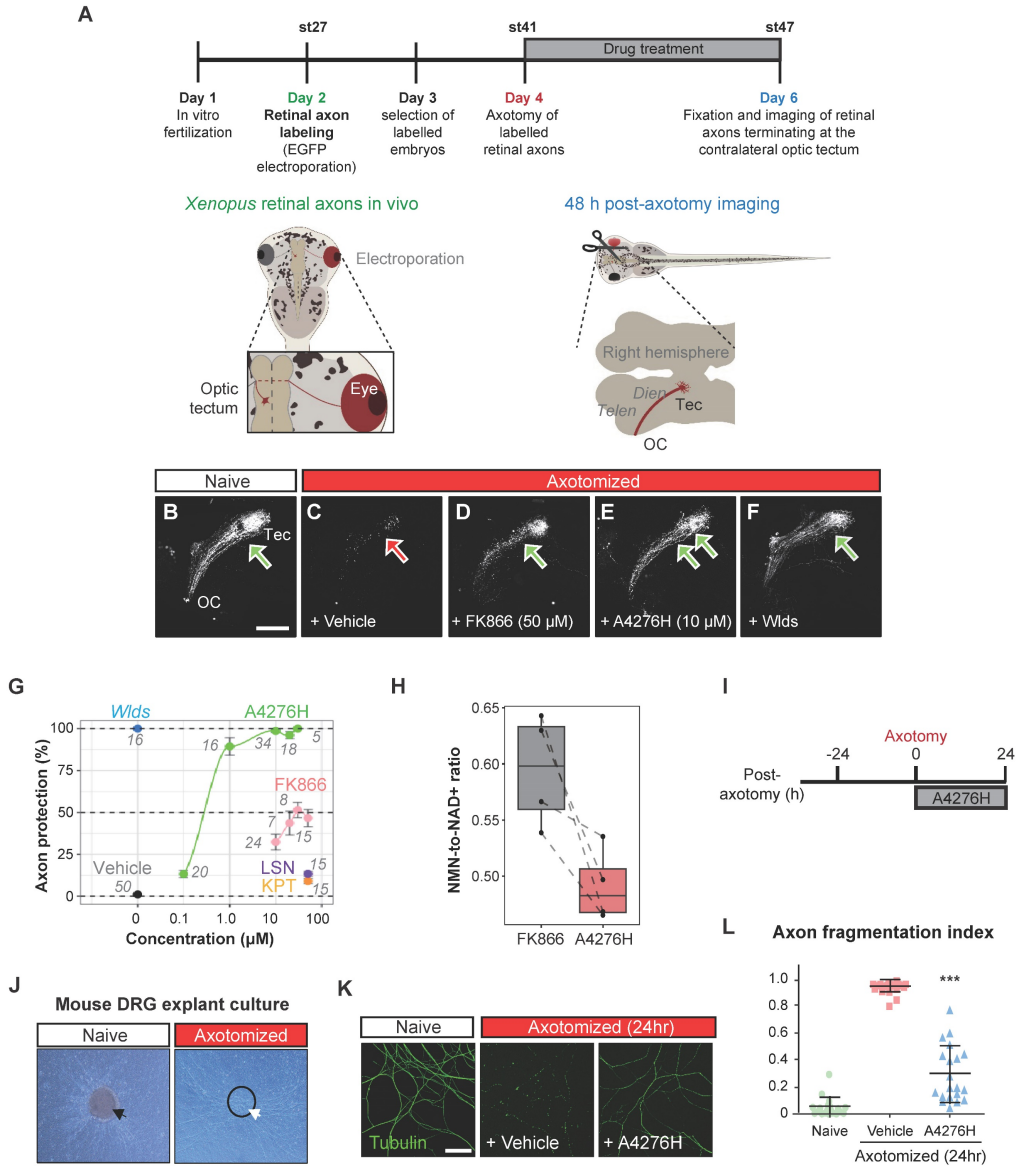
** A4276 delays Wallerian degeneration. (A)** Experimental scheme to assess Wallerian degeneration of *Xenopus* retinal axons *in vivo*. Retinal axons were labeled with EGFP through targeted electroporation, and Wallerian degeneration was induced by axotomy. A4276H, FK866, LSN3154567 (LSN), KPT-9274 (KPT) or vehicle were administered immediately after axotomy. Axon degeneration and survival were imaged 48 h post-axotomy. **(B-F)** Representative images of retinal axons terminating at the contralateral optic tectum treated with indicated concentrations of compounds at the end of experiments. **(G)** Quantification of axon protection effects of drug administration. The numbers indicate the number of animals per group. For A4276H and FK866, *p* < 10^-13^ in post hoc Tukey following one-way ANOVA for all comparisons except 0.1 µM, where *p* < 0.01, when compared to the vehicle. For LSN3154567, *p* < 0.05. For KPT-9274, *p* > 0.05. **(H)** Relative NMN-to- NAD+ ratios in tadpoles treated with FK866 or A4276H both at the same concentration (10 µM) and for the same duration as in Figure 6A. The ratio is normalized to that of untreated tadpoles. p < 0.016 in post hoc Tukey test following one-way ANOVA. **(I)** Experimental scheme to assess Wallerian degeneration of DRG neuronal axons in culture. **(J)** Representative images of DRG explant culture on a Boyden chamber, before (black arrow) and after (white arrow) scraping away the somata (dashed circle). **(K)** Immunocytochemical images of acetylated alpha-tubulin. **(L)** Comparison of the axon fragmentation indices. ***p < 0.001. Ordinary one-way ANOVA, followed by Bonferroni's multiple comparisons tests were used to calculate the significance; data represent mean ± SD (n = 20 each).

Notably, apart from their anti-neoplastic efficacy, NAMPT inhibitors have garnered attention for offsetting chemotherapy-induced adverse effects, especially as a remedy for Wallerian degeneration, the stereotyped degeneration of a severed distal axonal fragment, which is also dependent on SARM1, by hampering SARM1 activation 35. Based on the nature of A4276, an effective NAMPT inhibitor characterized by its profound tumor-suppressive effect not only as a single agent but also in combination with chemotherapy, we reasoned that it would also suppress SARM1-dependent axonal degeneration, further strengthening the therapeutic potential of A4276. We tested this hypothesis first with the retinal ganglion cells (RGCs), which are central nervous system neurons whose cell body is localized in the retina and whose axon projects to the visual centers of the brain in the thalamus and the midbrain. We used the amphibian model of *Xenopus tropicalis* because RGC axons in the brain can be selectively visualized by targeted electroporation of an EGFP-encoding plasmid into the retinal primordium, and Wallerian degeneration can be induced by severing the optic nerve 36. Severed axons almost completely disintegrate in two days by Wallerian degeneration, and this process is completely prevented by expressing Wlds, a gain-of-function mutant form of NMNAT1 that inhibits SARM1 activation and delays Wallerian degeneration 27, 37 (Figure 6A). We investigated whether A4276 and other NAMPT inhibitors, such as FK866, KPT-9274, and LSN3154567, could delay Wallerian degeneration and, if so, to what extent (Figure 6B-G). Remarkably, A4276H exhibited a much stronger effect in delaying Wallerian degeneration than any other NAMPT inhibitors, and its effect was comparable to that of Wlds overexpression (Figure 6E-F). Notably, the degree of protection conferred by A4276H treatment was not observed with any other NAMPT inhibitors, even at markedly higher concentrations (Figure 6G). Although FK866 delayed Wallerian degeneration in culture 38, our *in vivo* data revealed barely a partial delay of 48 h after axotomy compared to the effect of A4276H (Figure 6G), despite having similar effects in decreasing NAD+ concentration (Figure S8A). The axonal protection with A4276H was observed at 0.1 µM and reached its highest protective level at 10 µM (Figure 6G). Intriguingly, we observed that A4276H treatment results in a lower steady-state NMN-to-NAD+ ratio than FK866 treatment (Figure 6H). As SARM1 activation is triggered by an increase in the NMN-to-NAD+ ratio, we speculate that the different kinetics of A4276 in inhibiting NAMPT may result in a lower NMN-to-NAD+ ratio than other NAMPT inhibitors, thereby delaying the SARM1 activation.

To determine whether the axon-protective effect of A4276 could be observed in other types of neurons, we utilized mouse peripheral somatosensory neurons, the cell bodies of which are located in the dorsal root ganglia (DRG) (Figure 6I-J). We cultured DRG explants in the upper compartment of a Boyden chamber, allowing DRG neuron axons to grow through the porous membrane to the lower compartment, and induced Wallerian degeneration by removing DRG explants containing DRG cell bodies (Figure 6J). After 24 hours, we imaged healthy axons via immunostaining of stable microtubules (visualized by immunoreactivity against acetylated alpha-tubulin) following A4276H or vehicle treatment (Figure 6K-L). In accordance with the results observed in *Xenopus* RGC axons *in vivo*, A4276H prevented Wallerian degeneration of cultured DRG neuronal axons (Figure 6L). These results suggest that A4276 delays Wallerian degeneration not only in central nervous system neurons, but also in peripheral nervous system neurons in vertebrates.

### A4276 prevents chemotherapy-induced peripheral neuropathy

**Figure 7:**
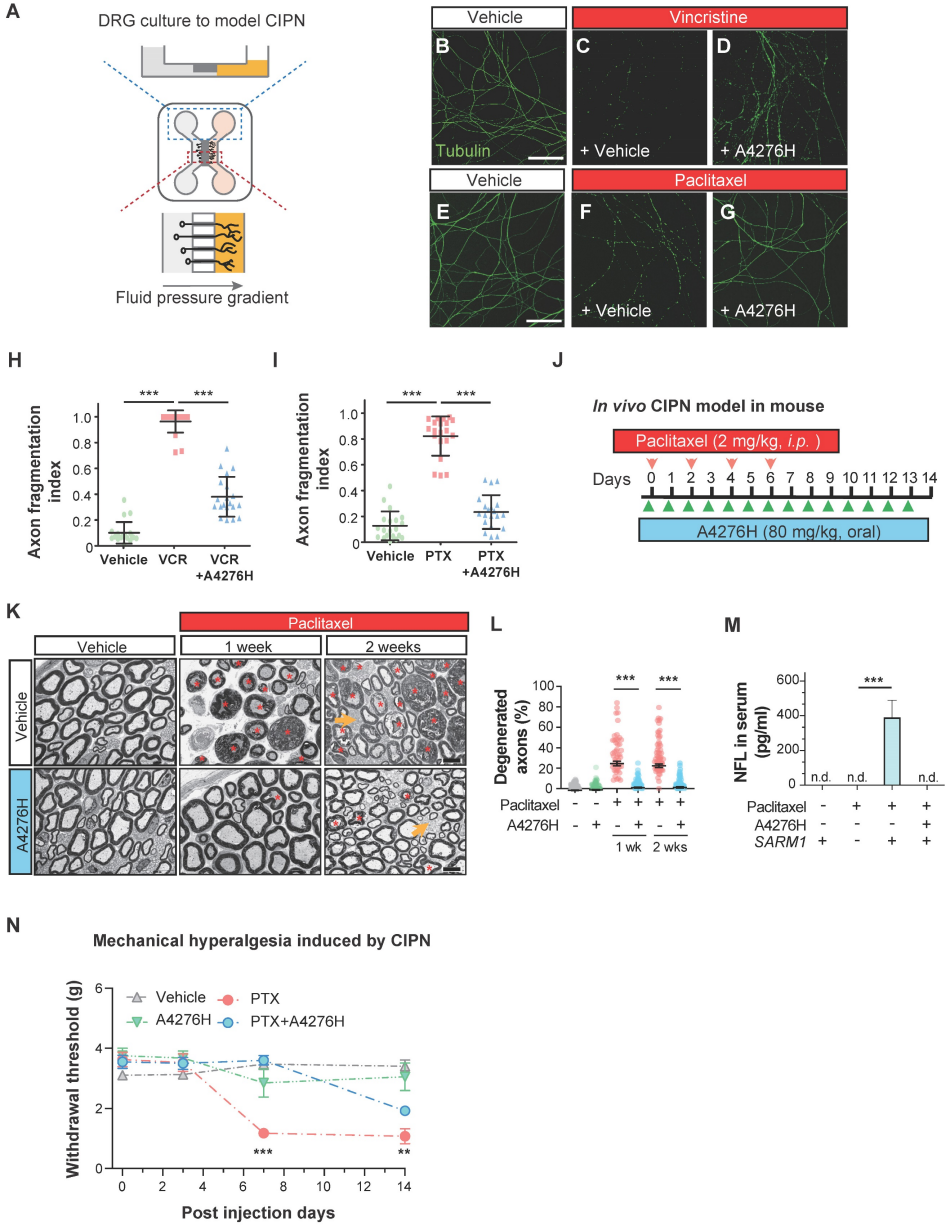
** A4276 prevents chemotherapy-induced peripheral neuropathy (CIPN). (A)** Experimental scheme for modeling CIPN in dissociated DRG cultures using a microfluidic device. **(B-G)** Representative immunocytochemical images of acetylated alpha-tubulin, treated with chemotherapeutics (vincristine 40 nM or paclitaxel 100 nM) with or without A4276H (2 µM). **(H-I)** Quantification of axon degeneration. ***p < 0.001; data represent mean ± SD (n=20 each). **(J)** Experimental scheme used to model CIPN in mice. Paclitaxel (2 mg/kg in 80% saline, 10% Kollophore EL, and 10% ethanol) were intraperitoneally injected for alternating four days. A4276H (80 mg/kg) was orally administered daily. **(K)** Transmission electron microscopy (TEM) images. Paclitaxel-induced nerve damage included atrophy (orange asterisks) and collapsed (red asterisks) axoplasms. Axons with these structural characteristics were counted as 'degenerated'. A4276H prevented degeneration of many axons. The unmyelinated axons (yellow arrows) did not degenerate in response to paclitaxel treatment. **(L)** Percentage of degenerated myelinated axons per image. ***p < 0.001 versus paclitaxel-only treatment. Data represent mean ± SD (n > 60 per group). **(M)** Serum NFL (neurofilament light) concentration on post-injection day 7, as determined using ELISA. ***p < 0.001 versus paclitaxel-only treatment; n = 3 each; n.d., not detected. **(N)** Mechanical sensitivity (von Frey test). Paclitaxel injection lowered the paw withdrawal threshold on day seven, which was prevented by A4276H. **p < 0.01 and ***p < 0.001 between paclitaxel-only treated group and paclitaxel plus A4276H-treated group; data represent mean ± SD (n = 4 each). **(H, I and L-N)** Ordinary one-way ANOVA followed by Bonferroni's multiple comparison tests were used to calculate the significance.

Chemotherapy-induced peripheral neuropathy (CIPN) is a significant clinical application of drugs that attenuate Wallerian degeneration. CIPN is a common adverse effect of chemotherapy, characterized by the development of painful neuropathy during drug administration and the degeneration of highly myelinated primary sensory axons, leading to a permanent loss of tactile sensation. As we observed a strong effect of A4276 in delaying Wallerian degeneration in DRG neuronal axons and CIPN, we investigated whether it could also prevent CIPN. The dissociated DRG neurons were cultured in a microfluidic device with fluidically separated somal and axonal compartments (Figure 7A). To model CIPN, we administered vincristine and paclitaxel, chemotherapeutic agents associated with CIPN, to the axonal compartment, which *in vivo* resides outside the blood-brain barrier and is vulnerable to the toxic effects of these agents. Co-treatment with A4276H prevented chemotherapy-induced axonal degeneration under these culture conditions (Figure 7B-I). A recent study showed that SARM1-dependent CIPN can also be delayed by nicotinamide riboside 5'-phosphoribosyltransferase (NAT5r), a NAMPT activator, perhaps by increasing NAD+ and decreasing the NMN-to-NAD+ ratio 39. Notably, NAT5r and A4276 showed similar degrees of protection against CIPN (Figure S8B-E), consistent with the proposition that inhibiting NAMPT in ways that keep the NMN-to-NAD+ ratio low may confer protection 27.

Having established the protective effect of A4276 in the *in vitro* model of CIPN, we further extended our investigation to an *in vivo* study using a mouse model. We administered paclitaxel intraperitoneally following a commonly used regimen in mice 40, and treated the mice with either A4276H or vehicle daily (Figure 7J). Fourteen days after the initial paclitaxel injection, we imaged the axonal ultrastructure of the sciatic nerve, which contained DRG axons, using transmission electron microscopy. Paclitaxel treatment resulted in specific degeneration of large myelinated axons (Figure 7K, red asterisks), including touch-sensitive peripheral DRG neuronal axons. However, treatment with A4276H significantly reduced this degeneration (Figure 7L). Consistent with our morphological analyses, we also observed that paclitaxel treatment led to an increase in the serum concentration of axonal cytoskeletal protein Neurofilament L (NFL) due to leakage from damaged axons. However, A4276H treatment almost completely blocked this increase in NFL (Figure 7M), indicating that A4276H successfully prevented axonal degeneration. In addition to damage to myelinated axons, CIPN also involves a transient increase in pain sensitivity due to the hypersensitization of lightly myelinated and unmyelinated nociceptive neuronal axons, which are spared in CIPN (Figure 7K, yellow arrows). Thus, we performed a von Frey test to measure allodynia, a lowered threshold of pain perception to such a degree that a normally innocuous stimulus induces an escape response (Figure 7N). In mice injected with paclitaxel, allodynia developed within one week, leading to a paw withdrawal response to light pressure that persisted throughout the injection period. In contrast, mice co-treated with A4276H displayed no such symptoms for one week and had significantly reduced pain hypersensitivity at the end of the injections (Figure 7N). Conclusively, our data clearly indicate that the co-administration of A4276 with chemotherapeutic agents can delay the development of CIPN, thus highlighting the potential utility of A4276 as a promising option for combination therapy in cancer treatment.

## Discussion

In this study, we developed and analyzed a novel NAMPT inhibitor, A4276, which exhibits remarkable anti-tumor activity against NAPRT-deficient EMT-subtype cancers. A4276 was characterized through biochemical studies, cellular research using various human cancer cell lines, and xenograft studies. Furthermore, we extended our investigation beyond the anti-tumor activity of A4276 to examine its ability to protect axons and delay CIPN development. Our findings suggest that A4276 has potential clinical applications not only as a single anti-tumorigenic agent but also for use in combination with chemotherapeutic drugs.

Owing to the heterogeneity of cancer, tailoring treatment regimens with agents that target tumors distinguished by a reliable response biomarker has emerged as a new paradigm in cancer treatment. Given the importance of the NAD+ supply in cancer cell survival and the SL relationship between two critical enzymes in NAD+ metabolism, NAMPT and NAPRT, we discovered a novel NAMPT inhibitor with improved biomarker selectivity. Our approach involved selecting a derivative of a hit compound that likely targeted cancer cells with NAPRT deficiency as a biomarker. A4276, an agent with greater selectivity than any other derivatives, was identified, and its selective cytotoxicity on NAPRT-depleted cancer cells was validated across multiple tumor types. Despite the similar NAPRT-negative cancer cell-targeting effects of two different NAMPT inhibitors, FK866 and KPT-9274, A4276 was noted for its wider therapeutic window, considering the discrepancy between the responses of NAPRT-positive and negative cancer cell lines (Figure 1C-D and, Figure S1B-C), along with its innocuousness in NAPRT-positive normal cell lines (Figure 3E and Figure S4). Moreover, the clear correlation between A4276 cytotoxicity and its response biomarker, NAPRT-deficiency was substantiated by the necessary and sufficient relationship between the NAPRT expression and A4276 resistance in NAPRT positive non-EMT cancer cell lines (Figure 4A-E, and Figure S5C). Overall, our study demonstrates not only the great potential of A4276 but also the feasibility of our approach for the optimization of a hit compound, which can enhance its selectivity and safety, accelerating the identification of a potent lead compound that targets cancer cells with an apparent biomarker.

One of the major obstacles to the clinical progress involving the first generation NAMPT inhibitors, FK866 and CHS828, was the occurrence of off-tumor toxicities, primarily thrombocytopenia and gastrointestinal symptoms 41-43. Consistent with this, in our study, FK866 and KPT-9274 were toxic to NAPRT-positive normal cells (Figure 3E, and Figure S4). To address these adverse effects, co-administration of NA with NAMPT inhibitors has been attempted to increase the therapeutic index (NCT02702492). However, the validity of this approach is still in question, as previous preclinical research with a NAMPT inhibitor, GNE-617, found that supplementation with 30 mg/kg or 100 mg/kg of NA impaired the anti-tumor efficacy of the compound 44. Interestingly, our xenograft study showed that A4276 inhibited the growth of tumors derived from HGC27 cells, even with NA supplementation at 100 mg/kg (Figure 5B). These findings highlight the potential utility of A4276 and suggest the possibility of widening its therapeutic window. Furthermore, we believe that our data support the addition of NA to maximize the safety of certain NAMPT inhibitors, thereby encouraging their clinical application.

Notably, NAPRT expression is often downregulated in several human cancers with EMT-like features, including gastric cancer (GC) 21, colorectal cancer (CRC), and pancreatic cancer (PAAD) (Figure 3B). EMT subtypes contribute to cancer progression by conferring metastatic capacity and greater resistance to therapy 45, 46. Therefore, our findings suggest that A4276 has the potential to eradicate treatment-resistant EMT-like cancer cells.

The development of effective treatment modalities for the tumor types we focused on (GC, CRC, and PDAC) is still an active area of ongoing research, as they are responsible for most cancer-related mortality 47-49. Currently, the standard of care for these cancers is either single- or multi-agent chemotherapy, which presents two significant challenges: resistance to therapy and adverse effects. To overcome these challenges, studies and clinical trials have focused on combination therapeutic strategies that combine chemotherapeutic agents with selected drugs designed to target molecularly defined tumor subsets. This approach has become a cornerstone of new treatment strategies for GC, CRC, and PDAC, enhancing the modest single-drug activity and lowering the risk of toxic side effects 47-49. Of particular interest is chemotherapy-induced peripheral neuropathy (CIPN), which is one of the most common and debilitating dose-limiting side effects of chemotherapy. This well-documented manifestation is caused by several chemotherapeutic drugs used widely for GC, CRC, and PDAC, including oxaliplatin and paclitaxel. However, no therapeutic option is currently available for patients with CIPN, despite the possibility of partial recovery with residual deficits 50, 51. SARM1, the driver of Wallerian degeneration underlying CIPN development, has been identified as a metabolic sensor controlled by the NMN/NAD+ ratio 25. These two metabolites compete for binding to the SARM1 ARM domain, with NMN binding having greater affinity and stimulating axon destruction. The generally abundant metabolite NAD+ acts in contrast to NMN when bound to SARM1 25, 52, 53. Although the basal ratio of NMN to NAD+ and the precise levels of each metabolite required for the regulation of mammalian SARM1 activity have yet to be fully elucidated 25, pioneering studies have corroborated the physiological effects of lowering NMN-to-NAD+ ratio and using NAMPT inhibitors in the treatment of axonopathies 35, 38. Based on these findings, we extended our research to explore the pharmacological potential of A4276 in preventing Wallerian degeneration. We confirmed that A4276H elicited protective effects against axonal degeneration, and at a remarkable level compared to other NAMPT inhibitors used in our study (FK866, KPT-9274, and LSN3154576) (Figure 6G). Furthermore, A4276H efficiently abrogated axonal fragmentation, alleviating not only Wallerian-like degeneration (Figure 6L) but also CIPN (Figure 7K-N) *in vivo*, even at a lower dose than that which hampered the growth of HGC27-derived xenograft tumors (Figure 5B). Supported by our findings (Figure S7) and studies indicating the synergistic anti-cancer effect of NAMPT inhibitors when treated with chemotherapeutic agents that cause peripheral neuropathies 24, 54, we conclude that the novel NAMPT inhibitor A4276 enhances chemotherapy and prevents neuropathy as a combinatorial cancer treatment against NAPRT-deficient cancers.

In the present study, we comprehensively analyzed and compared the efficacy of NAMPT inhibitors as anti-neoplastic agents and axonal protectors. In particular, comparative analyses with FK866, a relatively well-studied competitive NAMPT inhibitor in cancer and axonopathy, revealed the mechanism behind the A4276 efficacy. Excluding the possible involvement of secondary targets in the distinct response of certain NAPRT-positive cancer cell lines to FK866 and A4276 (Figure 4B-E, and Figure S5B), we propose that the optimal, yet not excessively stringent, NAMPT inhibitory effect of A4276 is a critical factor contributing to its ability to spare NAPRT-positive non-EMT cancer cell lines. The distinct binding mode (Figure 2F-G) and inhibition kinetic (Figure 4F) of A4276 compared to other NAMPT inhibitors may account for the discrepancies mentioned, leading to its higher selectivity against NAPRT-negative cancer cells. In line with this idea, we speculate that the kinetics of NAMPT inhibition by A4276 results in a lower NMN-to-NAD+ ratio and hence its superior ability to preserve damaged axons. To gain a more in-depth understandings in the distinct pharmacological effects of various NAMPT inhibitors, further investigation is required. This entails interrogating interactions with NAMPT in a biophysical and quantitative manner and assessing the kinetics of NMN and NAD+ depletion in different cell types. Given the complexity and heterogeneity in NAD+ consumption and production pathways among cells, along with the distinct dependence of tissues on NAD+ precursors 55, these studies may also offer valuable insights into the intricate regulation of NAD+ homeostasis.

Conclusively, the findings of this study have important translational implications. A4276 has not only demonstrated promise in the treatment of cancers with a clear biomarker, but also in preventing chemotherapy-induced axonal degradation. These results may help overcome major obstacles in the clinical management of cancer, such as targeting EMT-like cancers and preventing the development of CIPN.

## Materials and Methods

### Cell lines

Human NSCLC cell lines (H322, H661, H1155, H1993, H1975, H2030, H1299 and H2122) were kindly provided by Michael A. White and John D. Minna (UT Southwestern Medical Center, TX, USA). Gastric cancer cell lines, except for SK4 and Yonsei Cancer Center (YCC)-series cell lines, were purchased from the Korea Cell Line Bank (Seoul, Korea). The SK4 cell line was generously provided by Dr. Julie Izzo (MD Anderson Cancer Center, Houston, TX, USA), and the YCC-series cell lines were obtained from the Song-Dang Institute for Cancer Research, Yonsei University College of Medicine. All colorectal cancer cell lines except HGC27 were purchased from the Korea Cell Line Bank. The HGC27 cell line was obtained from the Riken cell line bank (Riken BioResource Research Center, Saitama, Japan). Normal cell lines including THLE-2, primary cardiomyocytes, and primary retinal epithelial cells were purchased from ATCC (Manassas, VA, USA), Promo Cell (Heidelberg, Germany), and Lonza (Basel, Switzerland), respectively. All pancreatic cancer cell lines were obtained from the Korea Cell Line Bank. NSCLC cell lines were cultured in RPMI-1640 medium supplemented with fetal bovine serum (Gibco, USA) and 1% penicillin-streptomycin (Invitrogen, USA) at 37°C in 5% CO_2_. Colorectal and pancreatic cancer cell lines were grown in RPMI-1640 medium supplemented with 10% fetal bovine serum (Gibco) and 1% penicillin-streptomycin (Invitrogen) at 37°C in 5% CO_2_. All normal cell lines were cultured according to standard protocols.

### Classifications of cancer cell lines

Cancer cell lines were classified into NAPRT-positive and NAPRT-negative groups by confirming NAPRT expression via immunoblot analysis. Ten colorectal cancer cell lines and eight pancreatic cancer cell lines were designated as EMT-like or non-EMT like based on the analysis of EMT signature genes as described in our previous report 21. Briefly, FPKM-normalized gene expression values of the cell lines were used and (FPKM + 1) values were Log2 transformed. The data were then subjected to unsupervised hierarchical clustering analysis of EMT signature genes with average linkages based on the Euclidean distance. The EMT gene signature comprises 149 upregulated and 161 downregulated genes obtained from EMT subtype gastric adenocarcinoma cohorts 56.

### Cell viability assay

Cell viability assays were conducted using the CellTiter-Glo assay system (Promega, Madison, WI, USA), according to the manufacturer's instructions. Briefly, cells were treated with compounds, including A4276, FK866 (B17LN09281, Uchem Meditech, Guangdong, Chian), and KPT-9274 (CS-5146, Chemscene, Monmouth Junciton, NJ, USA) for 72 h. Next, 10 μL of assay solution was added to each well, and the mixture was dispensed using a Multidrop dispenser (Thermo Fisher Scientific, Waltham, MA, USA). After 15 min of incubation at room temperature, luminescence was measured using a SpectraMax Paradigm microplate reader (Molecular Devices, San Jose, CA, USA). All values were normalized to those of DMSO controls. Drug-response curves were generated using the normalized viability of each agent, and the area under the curve (AUC) was calculated using the trapezoidal method. The 50% effective dose (ED50) was obtained by fitting a four-parameter log-based non-linear dose-response curve. In the 39 gastric cancer cell lines, 14 colorectal cancer cell lines, and 6 pancreatic cancer cell lines indicated in Figure 3C, the relative AUC values for A4276 were calculated based on nine-point dose-response curves. The cells were exposed to a range of A4276 concentrations (0-100 μM, half-log dilution series) for 72 h.

For the experiments to test the NAD+-biosynthetic pathway perturbation-dependent cytotoxicity of FK866 and A4276, cells were treated with the indicated compounds for 72 h and were subjected to the CellTiter-Glo assay. NAD+ (N0632, Sigma, Burlington, MA, USA), NAM (N0636, Sigma), NA (N4126, Sigma), or 2-Hydroxynicotinic acid (2-HNA) (247220250, Fisher Scientific) were utilized. The relative cell viability values were obtained by normalizing to the DMSO control group.

For the A4276-paclitaxel combination experiments, COLO320-HSR and HGC27 cell lines were treated with the drugs for 72 h, and a cell viability assay was conducted using the CellTiter-Glo assay system, as described above. The relative cell viability values were normalized to those of the DMSO control.

### Immunoblot analysis

The cells were harvested, washed with PBS, and lysed on ice for 15 min in radioimmunoprecipitation assay (RIPA) buffer (Sigma, R0278) containing a protease and phosphatase inhibitor cocktail (GenDEPOT, Katy, TX, USA). The lysates were centrifuged at 15,000 rpm for 20 min at 4°C, and the protein concentrations were determined using a Bradford assay (Bio-Rad, Hercules, CA, USA, 500-0006). Equal amounts of total protein were subjected to SDS gel electrophoresis and transferred to PVDF membranes (Bio-Rad). The membranes were blocked for one hour at room temperature (RT) before overnight incubation at 4°C with a primary antibody in buffer containing 0.1% Tween 20. The membranes were washed three times with Tween-PBS buffer and then incubated for an hour at RT with a secondary antibody diluted in blocking buffer containing 0.1% Tween 20. The membranes were washed three times with Tween-TBS for 10 min each. Immunoreactive bands were visualized using Pierce enhanced chemiluminescence (ECL) western blotting substrate (Thermo Fisher Scientific, 32106), SuperSignal West Pico PLUS chemiluminescent substrate (Thermo Fisher Scientific, 34578), and X-ray films (AGFA-Gevaert, Mortsel, Belgium). The following antibodies were used in this study: rabbit polyclonal anti-NAPRT antibody (1:1000, #NBP1-87243, Novus Biologicals), rabbit polyclonal anti-GRHL2 (1:2000, #PA5-28973, Thermo Fisher Scientific), rabbit monoclonal anti-PBEF/NAMPT antibody (1:1000, #86634, Cell Signaling Technology, Danvers, MA, USA), rabbit monoclonal anti-HSP90 antibody (1:1000, #4877, Cell Signaling Technology), rabbit monoclonal anti-zinc finger E-box-binding homeobox 1 (ZEB1) (1:1000, #3396, Cell Signaling Technology), rabbit monoclonal anti-Snail antibody (1:1000, #3879, Cell Signaling Technology), monoclonal anti-Vimentin antibodies (1:2000, #NB100-74564, Novus Biologicals or 1:1000, #5741, Cell Signaling Technology), rabbit polyclonal anti-E-cadherin antibody (1:1,000, #sc-7870, Santa Cruz Biotechnology, Dalas, TX, USA), mouse monoclonal anti-EpCAM antibody (1:1000, #sc-25308, Santa Cruz Biotechnology), and mouse monoclonal anti-beta-Actin antibody (1:5,000, #sc-47778, Santa Cruz Biotechnology).

### NAMPT activity assay

To investigate the effect of A4276 on NAMPT activity, an NAMPT Activity Assay Kit (Abcam, Cambridge, MA, USA ab221819) was used according to the manufacturer's instructions. In brief, NAMPT was incubated with various concentrations of A4276 in the presence of ATP, nicotinamide, nicotinamide mononucleotide adenylyltransferase 1 (NMNAT1), and phosphoribosyl pyrophosphate (PRPP) at 30°C for 60 minutes. After incubation, a mixture of water-soluble tetrazolium salts (WST-1), alcohol dehydrogenase (ADH), diaphorase, and ethanol was added to each sample and incubated for 30 min. The activity of NAMPT was then measured by determining the optical density (OD) at 450 nm on a microplate reader every 5 min for at least 30 min in the dark.

### NAMPT reactivation assay

The reactivation of inactivated NAMPT was monitored through the jump dilution method. At room temperature, NAMPT (200 nM) was incubated with excess A4276 (14 μM) for 60 minutes and then the concentration of A4276 was diluted to 70 nM by using assay buffer in the NAMPT Activity Assay Kit (Abcam, ab221819). The recovered NAMPT activity was measured as described above. NAMPT (200 nM) was incubated with DMSO (1.5 %) for 60 minutes and then the NAMPT activity was monitored.

### EMT score analysis

The EMT scores were calculated by subtracting the average log_2_-scale expression value of the 161 downregulated genes from that of the 149 upregulated genes included in the EMT gene signature. To enable an analysis involving multiple datasets with varying experimental designs, the calculated EMT scores were further subjected to Z-score normalization.

### Normalization and pre-processing of public gene expression data

The gene expression profiles of colorectal and pancreatic cancers were collected from multiple public cohorts. Variance-stabilizing transformations were conducted with raw counts using the R package DESeq2 (version 1.36.0) for TCGA-COAD, TCGA-READ, TCGA-PAAD, Severance colorectal cancer cohort, and GSE190826. The dataset GSE92921 was normalized in the CEL format with the robust multichip average method using the R package affy (version 1.74.0). For all other datasets, the expression values were downloaded as log_2_- normalized. To avoid duplicate entries per sample, we calculated the sums of the raw counts and mean expression values for other quantification methods.

### Correlation analysis for A4276 response versus NAPRT and NAMPT expression level in cancer cell lines

The transcriptomics dataset (v.23Q2) of lung, gastric, colorectal and pancreatic cancer cell lines were downloaded from DepMap portal (www.depmap.org). For the analysis, 19 cancer cell lines without gene expression data in the dataset were excluded from the 63 cancer cell lines where A4276 response was monitored. 44 cancer cell lines included 7 lung cancer cell lines (H322, H661, H1155, H1975, H2030, H1299, H2122), 23 gastric cancer cell lines (MKN1, HGC27, MKN74, SNU668, FU97, HS746T, AGS, NCIN87, IM95, KATO3, SK4, MKN45, SNU620, NUGC3, SNU5, SNU1, NUGC4, SNU216, SNU601, SNU16, OCUM1, SNU719, MKN7), 8 colorectal cancer cell lines (H716, SNUC2A, SNU81, SW480, LoVo, H508, SNUC4, HT29) and 6 pancreatic cancer cell lines (SNU410, MIA PaCa2, PANC1, Capan1, Capan2, SNU213).

### Analysis of NAMPT inhibition progress curve

The NAMPT Activity Assay Kit (Abcam, ab221819) was used to monitor NAMPT activity, following a similar procedure as described above. In detail, NAMPT was incubated with various concentrations of A4276 or FK866, along with ATP, nicotinamide, nicotinamide mononucleotide adenylyltransferase 1 (NMNAT1), and phosphoribosyl pyrophosphate (PRPP) at 30°C for 60 minutes. Subsequently, a mixture of water-soluble tetrazolium salts (WST-1), alcohol dehydrogenase (ADH), diaphorase, and ethanol was added to the samples and incubated for 30 minutes. NAMPT activity was then monitored every 5 minutes.

### Xenograft studies

All animal procedures were approved by the Institutional Animal Care and Use Committee (IACUC) of Yonsei University. Five-week-old female BALB/c-nu mice were purchased from SLC, Inc. (Shizuoka, Japan). Mice were housed in an individual ventilation cage system (IVCS) with a computerized environmental control system (Techniplast, Buguggiate, Italy). The room temperature was maintained at 22±2 °C with a relative humidity of 50±10%. The mice were allowed to adapt to their laboratory housing environment for at least one week before the experiments. Five-week-old mice were subcutaneously injected with 5x10^6^ HGC27 cells. Once the tumor volume reached 150 mm^3^, the mice were randomly divided into eight groups, and 6-7 mice per group were orally administered with various doses of A4276H (HCL salt form of A4276), KPT-9274, or the vehicle for 21 d. For NA co-administrated groups, 100 mg/kg of NA was added together with the compounds. Tumors were measured (length × width) every 2-3 d, and the weights of the mice were measured every 1-2 d from the first day of drug treatment. Tumor volume was calculated using the following formula: 0.5 × major axis × (minor axis)^2^. Further analyses were conducted using the data obtained from mice with a tumor volume of at least 100 mm^3^ on the first day of drug administration.

### NAD/NADH assay

To monitor the NAD(H) levels (indicated as total NAD) in cell lines, the NAD/NADH-GLO^TM^ assay kit (Promega, G9071) was used. After 48 h of seeding 0.5 x 10^4^ cells per well into a 96-well culture plate, each compound was treated for 24 h. Subsequently, the media was replaced to 50 µl of PBS, followed by the addition of 50 µl NAD/NADH-Glo^TM^ Detection reagent. The total amount of NAD present in resulting 100 µl of solution in each well was directly measured according to the manufacturer's instructions.

NAD(H) levels in the tumor xenografts were quantified using an NAD/NADH colorimetric quantification kit (BioVision, Milpitas, CA, USA #K337-100) according to the manufacturer's instructions. Briefly, 20 mg of tissue sample was washed with ice-cold PBS and extracted with 400 μl of NAD/NADH extraction buffer. The total NAD+ absorbance was measured at 450 nm using a SpectraMax paradigm microplate reader and normalized to the total protein concentrations, which was determined using Bradford assay.

### Combination index calculation

Combination index (CI) was calculated based on the Loewe additivity model and median-effect principle, which refers to the Chou-Talalay method using the SiCoDEA tool 57. The relative viability data of COLO320-HSR and HGC27 assessed after 72 h of exposure to various combination concentrations of paclitaxel and A4276 were used to calculate the CI values. Subsequently, the CI values were plotted against the fractional affected (Fa) value. The Fa value was obtained by subtracting the fraction unaffected value (Fu = viable cell percentage / 100) from 1.

### *Xenopus* experiments

Labeling of the *Xenopus* retinal axons and imaging of their degeneration were performed as previously described 58. Briefly, stage 27-28 *Xenopus* embryos were anesthetized and a plasmid encoding EGFP was transfected into the optic vesicle via targeted electroporation. At stage 41, when the retinal ganglion cell axons had reached the optic tectum, the optic nerve originating from the electroporated retina was severed. The drug was administered at the time of axotomy throughout the experiments. The EGFP-positive retinal axons terminating at the contralateral optic tectum were imaged using a laser scanning confocal microscope (LSM700, Zeiss, Germany).

### Quantification of delay in Wallerian degeneration in *Xenopus*

After axotomy, retinal axons undergo stereotypical steps of synchronous degeneration, which can be clearly distinguished at 24-h intervals, with approximately 50%, 15%, and 0% axonal fragments remaining at 24, 48, and 72 h post-axotomy, respectively 58. We quantified the effects of the drugs on Wallerian degeneration by comparing images of drug-treated axons at 48-h post-axotomy with those of the normal course of Wallerian degeneration. If a drug completely prevented axon degeneration at 48-h post-axotomy, an axon protection index of 100% was assigned. If the drug delayed Wallerian degeneration by 24 h, an axon protection index of 50% was assigned. If the drug had no effect on the percentage of surviving axons, the axon protection index was defined as 0%. Using this approach, WldS over-expression conferred 100% protection, whereas vehicle treatment conferred 0% protection. Treatment with FK866 at concentrations ranging from 10 µM to 50 µM resulted in approximately 50% protection, whereas treatment with A4276H at 1 µM or higher concentrations conferred 100% protection.

### Measurement of NAD^+^ concentration in *Xenopus* embryos

Axotomy was performed on stage 41 *Xenopus* embryos, and the drug (vehicle, FK866, A4276H, KPT-9274, LSN3154567) was administered at 10 μM for 48 h. NAD+ concentration was measured using NAD/NADH-Glo Assays (Promega, G9071) and a microplate reader (EG&G Berthold, Centro XS3 LB960). All experiments were conducted with at least three independent biological replicates.

### Measurement of NMN-to-NAD+ ratios in *Xenopus* embryos

Drug (vehicle, FK866, or A4276H) was administered at 10 μM for 48 h. Tadpole samples were collected in three groups (WT, FK866, A4276H), 50 mg each in a 1.5 ml tube (n=4). Nicotinamide mononucleotide (NMN), Nicotinamide adenine dinucleotide (NAD+) were extracted from 500 µL of pre-cooled (-20 °C) 80% methanol with internal standard (ISTD) 2-chloroadenosine. ISTD was added to all samples at the concentration of 40 ng/mL for quality control 59, 60. Methanol and ISTD were added to the samples, followed by vortex mixing for 4 min and centrifugation for 10 min at 16,000 ×*g* and 4 °C. Afterward, 500 µL of chloroform was added to the supernatants and centrifugation was performed for 10 min at 16,000 ×*g* and 4 °C. Chloroform extraction was repeated, and supernatants were dried using a speed vacuum concentrator with a cold trap (CentriVap Cold Traps, Labconco, Kansas City, MO, USA). Each dried sample was reconstituted in 100 µL of HPLC grade water and centrifuged for 10 min at 16,000 ×*g* and 4 °C. The supernatants were transferred to vials for liquid chromatography-mass spectrometry analysis.

Extracted samples were injected into ACQUITY UPLC BEH Amide 1.6 μm C18 130Å, 100 mm × 2.1 mm column (Waters, Milford, MA, USA) and separated over 6 min (0.6 mL/min) using a gradient of solvent B at 40 °C. Solvent B was 0 min, 80% B; 1 min, 80% B; 2 min, 5% B; 3.5 min, 5% B; 3.51 min, 80% B; 6 min, 80% B. Solvent A was composed of 20mM ammonium acetate in water (pH 3.2 with formic acid) and B was acetonitrile (100%). Injection volume was 2 µL. Polarity was set at positive mode. Liquid chromatography system was coupled to a SCIEX QTRAP 5500+ (AB SCIEX, Framingham, MA, USA) with Nexera series LC-40 (Shimadzu Corporation, Kyoto, Japan).

Single reaction monitoring (SRM) analysis was performed. Declustering potential (DP), collision energy (CE), and collision cell exit potential (CXP) were optimized for the target compound. Each target was optimized with a total of two transitions. One transition was used for quantification and the other for qualification. MRM transitions, DP, CE, CXP are summarized in Table S2. The MS parameters were collision gas, 8 psi; curtain gas, 35 psi; ion source gas 1, 40 psi; ion source gas 2, 60 psi; ion spray voltage, 4500 V; and source temperature, 550 °C. The data acquisition and processing were carried out using Analyst (AB SCIEX, version 1.7.3 HotFix 1) and SCIEX OS (AB SCIEX, version 3.0.0.3339) software. Liquid chromatography—mass spectometry experiments were performed by the Prometabio Research Institute (Prometabio Co., Ltd. Hanam-si, Gyeonggi-do, Republic of Korea).

### Mouse DRG explant culture

The dorsal root ganglia (DRG) were aseptically removed from postnatal mice, and each ganglion was cut into several pieces. Cell culture inserts (1 µm pore size, Falcon), also known as the Boyden chamber, adequate for a 6-well plate, were coated with poly-L-lysine and Laminin (both from Sigma). The inserts were placed in a 6-well plate and each well was filled with 2 mL of Neurobasal A medium supplemented with 2% B-27, 0.5% glutamine, and 1% antibiotic antimycotic (all purchased from Thermo Fisher) with 50 ng/mL of NGF (Sigma). DRG explants were placed on the insert and allowed to grow axons through the pores on the bottom surface of the insert, which was facilitated by the NGF in the lower compartment (Figure 6J). To induce axotomy and Wallerian degeneration, the cell bodies on the top side of the insert were scraped away using a scraper. In some wells, 2 μM A4276H was applied 24 h before axotomy as pretreatment. Medium containing A4276H was changed daily.

### Mouse dissociated DRG neuronal culture

The dorsal root ganglia (DRG) were aseptically removed from postnatal mice and treated with 0.25% trypsin at 37℃ for 15 min, after which the trypsin was neutralized with fetal bovine serum. The DRG were centrifuged at 3000 rpm for 5 min and resuspended in Neurobasal A medium supplemented with 2% B-27, 0.5% glutamine, and 1% antibiotic antimycotic (all purchased from Thermo Fisher) with 10 ng/mL of NGF (Sigma). The DRG was then dissociated into single cells through trituration with flame-polished glass pipettes and pre-plated on an uncoated Petri dish to remove adherent cells. After 2 h, loosely attached neurons were dislodged by gentle pipetting and plated into one compartment (i.e. “somal” compartment) of a microfluidic device that had been precoated with poly-L-lysine and Laminin (Sigma) (Figure 7A). A higher concentration of NGF (50 µM) was added to the other compartment (i.e. “axonal” compartment) to induce the growth of axons from the somal compartment through the microgrooves into the axonal compartment. Additionally, a slightly higher volume of medium was added to the somal compartment to create a microfluidic environment that prevents axonal-to-somal flow. Vincristine 61 and paclitaxel 62 were added to the axonal compartments to induce axonal degeneration. Cells were pretreated with A4276 (2 μM), FK866 (5 µM), or NAT5r (5 µM, BLDpharm, Shanghai, China) 24 h prior to exposure to these chemotherapeutic drugs. Vincristine was administered at a concentration of 40 nM for 24 h, followed by treatment with 100 nM paclitaxel for 48 h.

### Quantification of axon degeneration in culture

The cultures were fixed at specific time points using 4% paraformaldehyde in PBS. Stable microtubules in the axons were visualized using immunocytochemistry with an antibody against acetylated alpha-tubulin (Abcam, ab125356) and an Alexa Fluor 488-conjugated secondary antibody (Thermo Fisher, A-21206). Samples were immuno-stained and imaged under identical settings for quantitative analyses. The axon fragmentation index was defined as the proportion of the lost axonal length. Specifically, traces of axons in an image were manually delineated as segmented lines. The channel for acetylated alpha-tubulin was thresholded to generate a binary copy of the same image on which traces of axons were superimposed. Then axon fragmentation index for the entire image was calculated as [the sum of the lengths of axons not stained with acetylated alpha-tubulin] divided by [the sum of all axonal traces]. All experiments were performed with at least three independent biological replicates.

### Induction of Wallerian degeneration in mice

Mice were anesthetized using Zoletil, the sciatic nerve was transected, and the wound was sutured. After surgery, the mice were euthanized at 1, 3, or 7 d, and serum and sciatic nerve fragments distal to the injury were collected for analysis.

### Induction of paclitaxel-induced peripheral neuropathy in mice

Paclitaxel (2 mg/kg) dissolved in 80% saline, 10% Kolliphor EL, and 10% ethanol were injected intraperitoneally for four alternating days 40. The von Frey test was performed on post-injection days 3, 7, and 14 to measure hyperalgesia. On day 14, the mice were euthanized and both serum and sciatic nerve fragments were collected for analysis.

### Imaging of the sciatic nerve and quantification of axon degeneration

The sciatic nerve was fixed, processed, and imaged using transmission electron microscopy (TEM) at the Zeiss-Yonsei imaging facility. Myelinated axons, both healthy and degenerated, were manually counted for quantitative analysis.

### Measurement of NFL concentration in serum

Serum NFL concentration was measured using an NF-Light ELISA kits (UmanDiagnostics, Sweden).

### Von Frey test

The von Frey test was performed on post-injection days 3, 7, and 14, according to the protocol provided by the Jackson Laboratory Mouse Neurobehavioral Phenotyping facility. Prior to the test, the mice were habituated to a wire grid for 10 min, and then each hind paw was stimulated twice for each trial.

### Administration of A4276

An oral administration of A4276 (80 mg/kg) dissolved in 0.5% methylcellulose and 0.2% Tween 80 was given to 7-week-old mice. All experimental procedures were performed in accordance with protocols approved by the Yonsei University College of Medicine IACUC.

### Purification, crystallization, data collection, and structure determination

Recombinant NAMPT proteins (GenScript, Piscataway, NJ, USA) were purified after buffer exchange. The PBS buffer (pH 7.4) containing 10% glycerol was changed to S-buffer consisting of 25 mM Tris pH 8.0 and 2 mM DTT. Next, NAMPT in S-buffer was loaded onto a HiTrap Q FF column (Cytiva, Chicago, Illinois, USA) pre-equilibrated with S-buffer and eluted using a 0 - 1 M NaCl gradient in the same buffer. Eluted fractions were concentrated and loaded onto Superdex 75 HR 16/60 column (Cytiva). Purified NAMPT in a buffer containing 25 mM Tris-HCl pH 8.0 and 2 mM DTT was concentrated to approximately 10 mg/mL for crystallization. The microbatch crystallization set up 63, 64 was used to grow crystals at 10°C in droplets under a thin layer of Al's oil that contained 1 µL protein solution and 1 µL precipitant solution of 25% (w/v) polyethylene glycol (PEG) 3350, 0.1 M sodium phosphate pH 7.0, and 0.2 M sodium chloride. A 2.09 Å resolution data set was collected from a flash-cooled crystal at 100 K using a DECTRIS EIGER X 9M detector installed at beamline 5C (Pohang Light Source, Republic of Korea). Diffraction data were processed and scaled using XDS and XSCALE 65. The crystal belonged to space group *P*2_1_2_1_2_1_ (a = 97.56 Å, b = 116.69 Å and c = 195.41 Å) with four molecules in the asymmetric unit (Table S3). Molecular replacement was performed with *Phaser* 66, using the structure of NAMPT apo (PDB entry 2e5b) as a search model. Several refinement and manual refitting rounds were performed using *Phenix* 67 and *Coot* 68, respectively. The final model (R_work_/R_free_ = 20.41/23.13 %) consisted of residues 8 - 42 and 53 - 483 of NAMPT, four A4276 molecules, 710 water molecules, and 12 phosphate molecules (Table S3). Atomic coordinates and structure factors have been deposited in the Protein Data Bank under the accession code 8ivu.

### Statistical analysis

Graphs were obtained using GraphPad Prism and R. All statistical tests were performed using the R statistical software (ver. 4.0.3). We performed an unpaired t-test, one-way ANOVA test with Tukey's multiple comparison test, and two-way ANOVA, as stated in the corresponding legends of the article. *P*-values are denoted as follows: * < 0.05, ** < 0.01, *** < 0.001, and not significant (ns) ≥ 0.05. R version 4.0.3 [(2020-10-10), platform: x86_64-w64-mingw32 (64-bit), and operating system: Windows 10 × 64].

## Supplementary Material

Supplementary figures and tables.

## Abbreviations

SL: synthetic lethality
NAMPT: nicotinamide phosphoribosyltransferase
NAD+: nicotinamide adenine dinucleotide
NAPRT: nicotinic acid phosphoribosyltransferase
EMT: epithelial-to-mesenchymal transition
FAO: fatty acid oxidization
SARM1: sterile alpha and TIR motif containing 1
PARP: Poly ADP-ribose polymerase
NAM: nicotinamide
PH: preiss-handler
NA: nicotinic acid
PROTAC: proteolysis-targeting chimera
NMN: nicotinamide mononucleotide
NMNAT2: nicotinamide mononucleotide adenylyltransferase 2
CIPN: chemotherapy-induced peripheral neuropathy
AUC: area under the curve
ED50: 50% effective dose
IC50: half-maximal inhibitory concentration
Tyr: tyrosine
Phe: phenylalanine
Asp: aspartic acid
Ser: serine
Ala: alanine
His: histidine
Val: valine
Ile: isoleucine
Arg: arginine
GC: gastric cancer
CRC: colorectal cancer
PDAC: pancreatic ductal adenocarcinoma
ZEB1: zinc finger E-box-binding homeobox 1
GRHL2: grainyhead-like transcription factor 2
EpCAM: epithelial cell adhesion molecule
RPE: retinal pigment epithelium
PAK4: p21-activated kinase 4
2-HNA: 2-hydroxynicotnic acid
PK: pharmacokinetics
hERG: human ether-à-go-go-related gene
CI: combination index
RGC: retinal ganglion cell
WLDs: wallerian degeneration slow
DRG: dorsal root ganglia
NAT5r: nicotinamide riboside 5'-phosphoribosyltransferase
NFL: neurofilament light chain
