# Supplementary material for: Discovery of a novel NAMPT inhibitor that selectively targets NAPRT-deficient EMT-subtype cancer cells and alleviates chemotherapy-induced peripheral neuropathy
Source: Theranostics. 2023 Sep 11;13(14):5075–98. doi: 10.7150/thno.85356 (PMC10526665; doi:10.7150/thno.85356)
Supplement: Supplementary file 1 — Supplementary figures and tables. [file thnov13p5075s1.pdf]

# Supplementary Materials

**Title:** Discovery of a novel NAMPT inhibitor that selectively targets NAPRT-deficient EMT-subtype cancer cells and alleviates chemotherapy-induced peripheral neuropathy

**Authors:** Minjee Kim[1,2,#], Hyeyoung Kim[1,3,#], Bu-Gyeong Kang[4,#], Jooyoung Lee[2,5,#], Taegun Kim[6,#], Hwanho Lee[1,2], Jane Jung[1,3], Myung Joon Oh[1,2], Seungyoon Seo[7], Myung-Jeom Ryu[2], Yeojin Sung[1,2], Yunji Lee[1,2], Jeonghun Yeom[7], Gyoonhee Han[6,*], Sun-Shin Cha[4,*], Hosung Jung[1,3,*], Hyun Seok Kim[1,2,5,*]

**Affiliations:**

1. Graduate School of Medical Science, Brain Korea 21 Project, Yonsei University College of Medicine, Seoul, 03722, Republic of Korea
2. Department of Biomedical Sciences, Yonsei University College of Medicine, Seoul, 03722, Republic of Korea
3. Department of Anatomy, Yonsei University College of Medicine, Seoul, 03722, Republic of Korea.
4. Department of Chemistry and Nanoscience, Ewha Womans University, Seoul, 03760, Republic of Korea
5. Checkmate Therapeutics Inc., Seoul, 07207, Republic of Korea
6. Department of Biotechnology, College of Life Science and Biotechnology, Yonsei University, Seoul 03722, Republic of Korea.
7. Prometabio Research Institute, Prometabio Co., Ltd. Hanam-si, Gyeonggi-do 12939, Republic of Korea

[#] Equal contribution

[*]**Correspondence:** hsfkim@yuhs.ac (lead contact), hjung@yuhs.ac, chajung@ewha.ac.kr, gyoonhee@yonsei.ac.kr

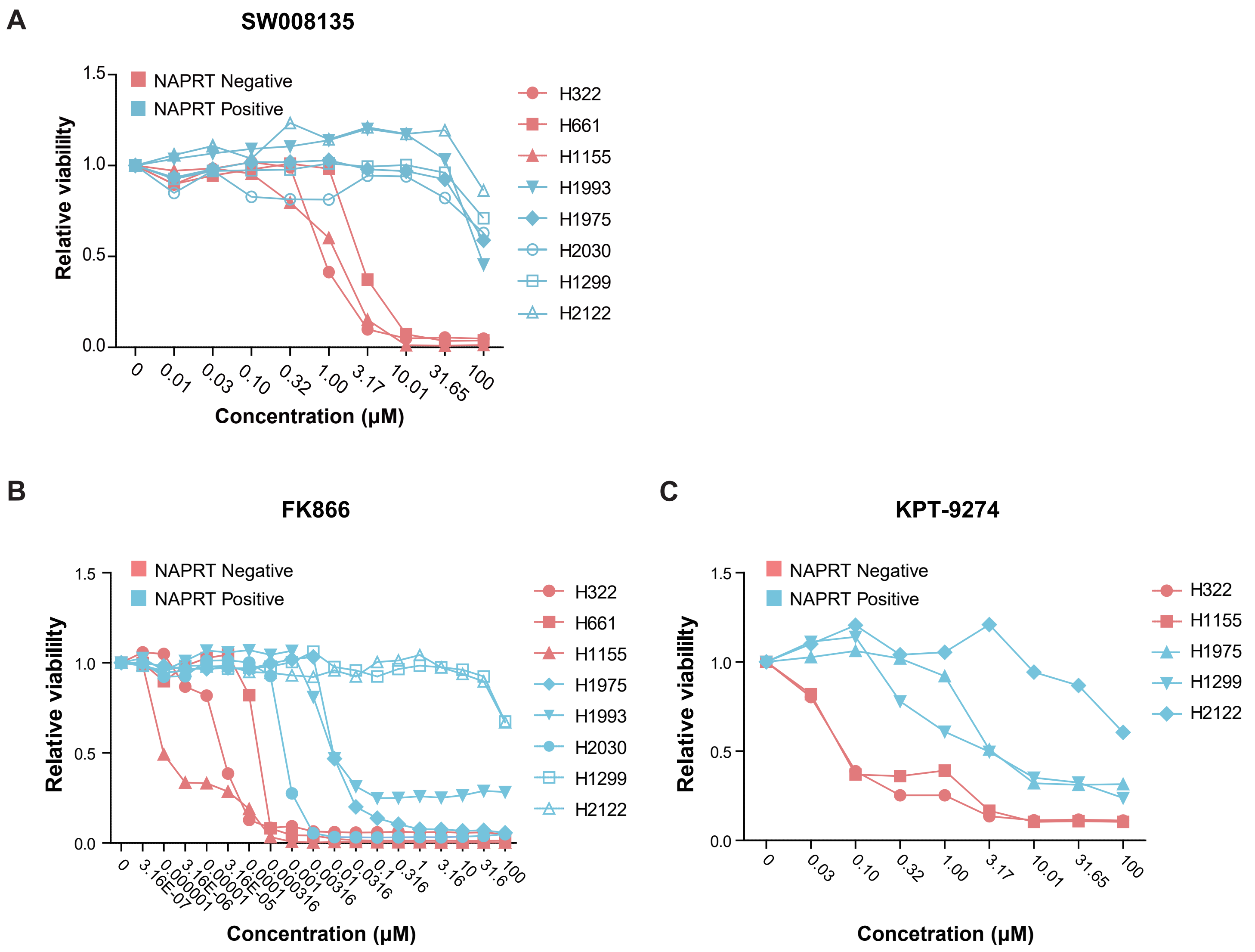


**Figure S1. The different responses of a panel of lung cancer cell lines against NAMPT inhibitors. (A, B, C)** Dose-response curves for indicated lung cancer cell lines after 72 hours of exposure to SW008135 **(A)** FK866 **(B)** and KPT-9274 **(C)**, respectively. NAPRT-positive cell lines are presented as light blue and NAPRT-negative as light red. The relative area under curve (AUC) values and Log [ED50] values for each compound in Figure 1D were calculated based on the dose-response-curve presented in (B) and (C).

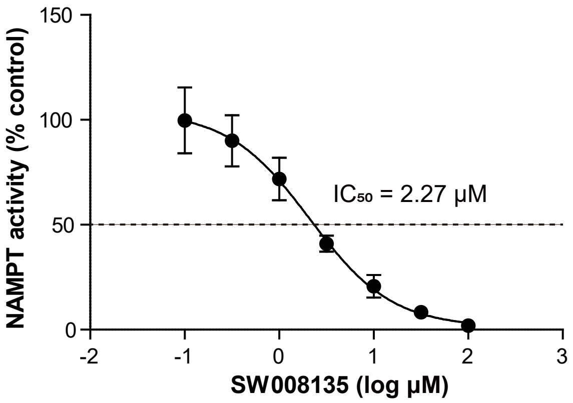


**Figure S2. IC50 value of SW008135.**

The NAMPT enzyme activity assay showing SW008135 concentration-dependent inhibition of NAMPT.

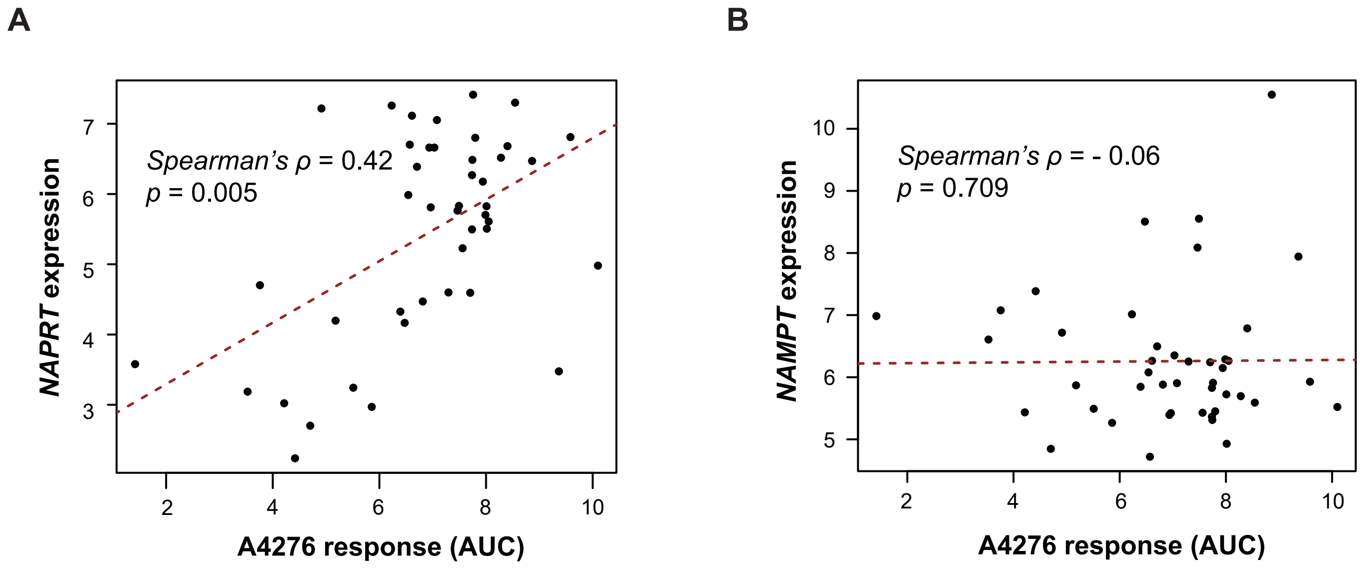


**Figure S3. The response against A4276 shows a strong inverse correlation with NAPRT expression, but not with NAMPT expression, in various cancer cell lines.**

**(A, B)** The correlation between the sensitivity to A4276, represented by the area under the curve values, and the expression level of the indicated genes. Out of the 63 cancer cell lines used in Figure 1B and 3C, 44 were included in the analysis, as they have expression data available in the DepMap dataset (See methods for details). Statistical significance of the correlation was analyzed using Spearman's rank correlation test.

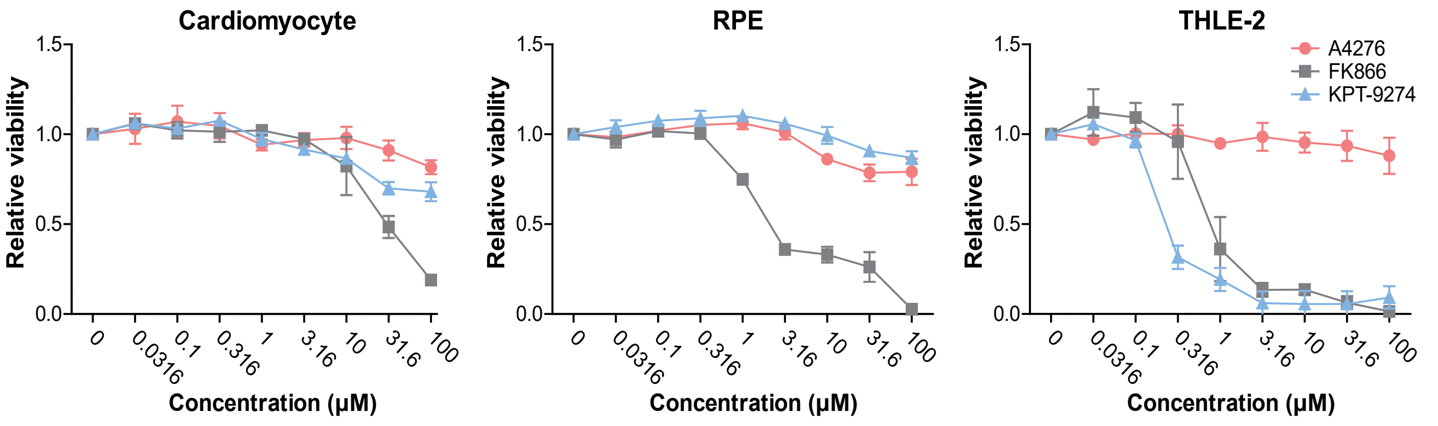


**Figure S4. The different responses of normal cell lines against NAMPT inhibitors.**
Dose-response curves for indicated normal cell lines after 72 h of exposure to each NAMPT inhibitor at various concentrations. Following data have been utilized for the calculation of relative AUC, presented in Figure 3E. Data represent mean ± SD (n = 3).

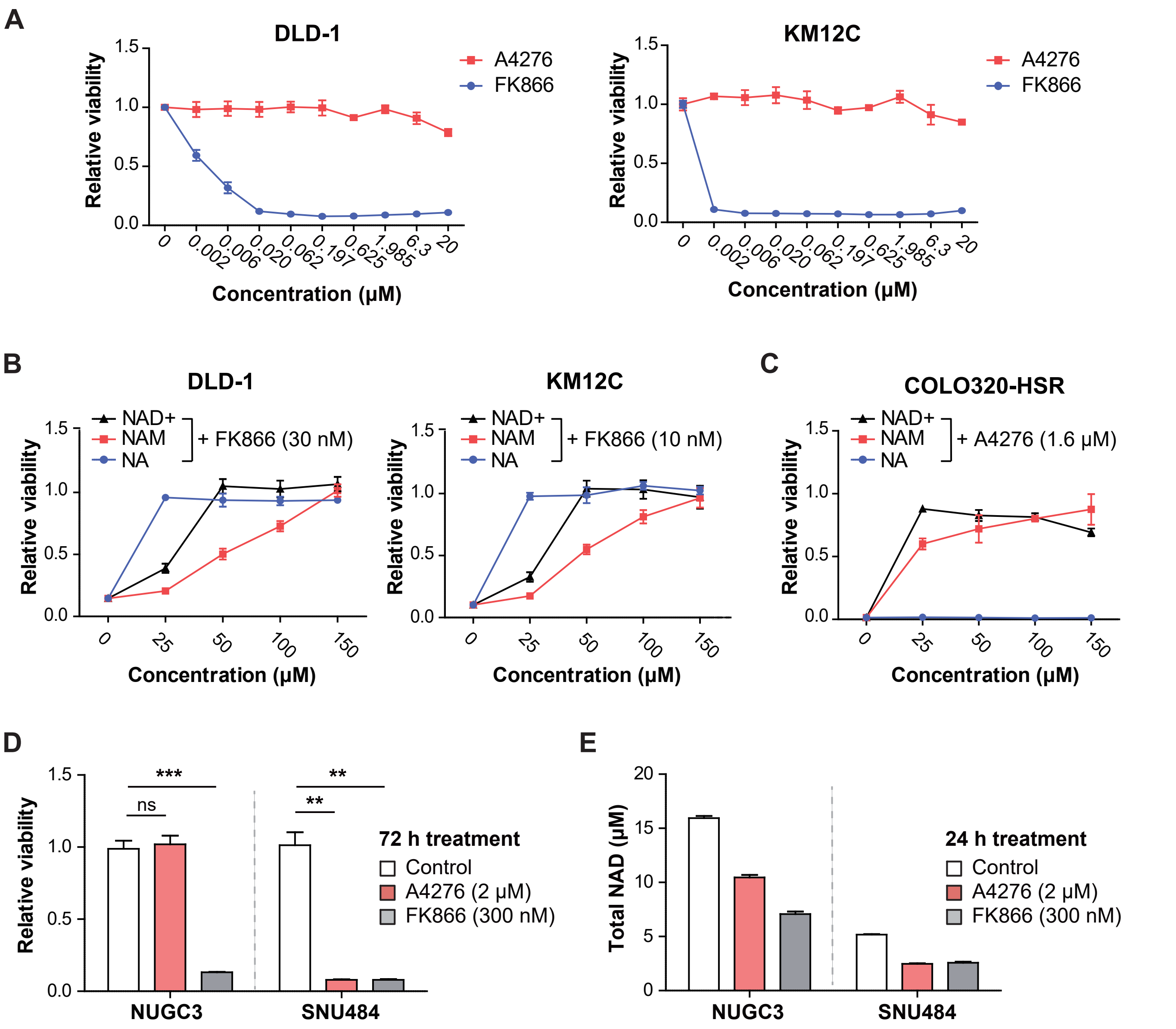


**Figure S5. Differential effects of FK866 and A4276 in various cancer cell lines.**
**(A)** Dose-response curves for two NAPRT-positive non-EMT cancer cell lines (DLD-1 and KM12C) after 72 h of exposure to a various concentration of A4276 or FK866. **(B)** Relative viability of each cell line was assessed after 72 h incubation with FK866 in addition to NAD+, nicotinamide (NAM) and nicotinic acid (NA) at the indicated concentrations. **(C)** Relative viability of the NAPRT-negative EMT cancer cell line COLO320-HSR was assessed after 72 h treatment of A4276 in combination with NAD+, nicotinamide (NAM), and nicotinic acid (NA) at the indicated concentrations. **(D)** Relative viability of each cell line was assessed after 72 h treatment with indicated concentration of compounds. ***$p < 0.001$, **$p < 0.01$, and not significant (ns). Student's t-test was used for the comparison. **(A-D)** Data represent mean ± SD (n = 3). **(E)** The amount of total NAD of indicated cells was assessed after 24 h treatment with each compound (See methods for details). ***$p < 0.001$. Significance of the interaction between cell type and treatment was tested by two-way ANOVA. Data represent mean ± SD (n = 2).

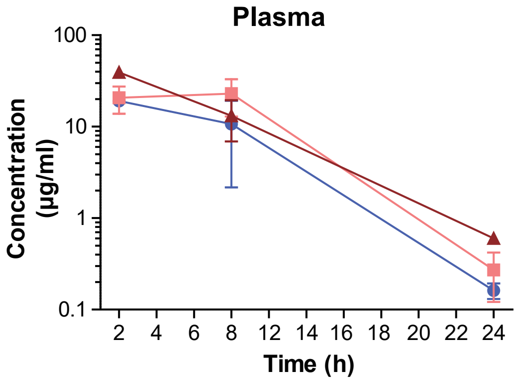


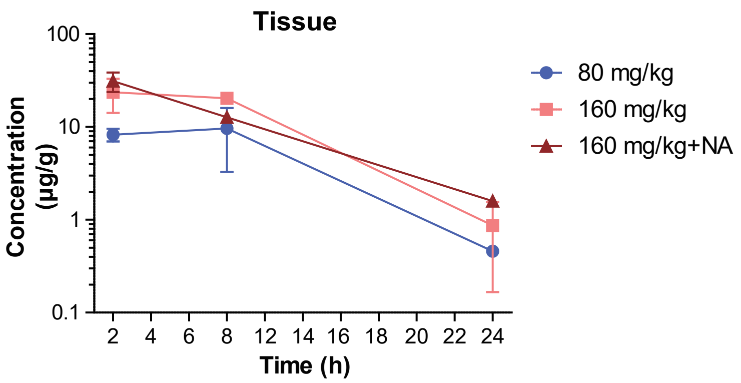


| Parameter | 80 mg/kg | 160 mg/kg | 160 mg/kg + NA |
|---|---|---|---|
| $T_{max}$ (h) | 2.00 ± 0.00 | 8.00 ± 0.00 | 2.00 ± 0.00 |
| $C_{max}$(µg/ml) | 19.73 ± 0.81 | 22.94 ± 0.81 | 39.19 ± 2.84 |
| $AUC_{last}$ (µg*h/ml) | 195.38 ± 96.68 | 337.16 ± 138.56 | 306.29 ± 79.63 |

| Parameter | 80 mg/kg | 160 mg/kg | 160 mg/kg + NA |
|---|---|---|---|
| $T_{max}$ (h) | 5.00 ± 4.24 | 5.00 ± 4.24 | 2.00 ± 0.00 |
| $C_{max}$(µg/ml) | 11.64 ± 3.48 | 25.48 ± 6.83 | 31.20 ± 7.43 |
| $AUC_{last}$ (µg*h/ml) | 142.51 ± 64.59 | 324.73 ± 38.93 | 219.66 ± 50.57 |
| T/P ratio | 0.73 | 0.96 | 0.72 |

**Figure S6. Pharmacokinetic profiles of A4276H.**

The graphs represent the concentration of A4276H measured *in vivo* in the plasma (left) and tumor tissue (right) at 2, 8, and 24 h after oral administration. Data represent mean ± SD (n = 2 each). The pharmacokinetic parameters of A4276H are listed in the tables below the graphs.

$C_{max}$: maximum concentration; $T_{max}$: time of $C_{max}$; $AUC_{last}$: area under the curve to the last measurable concentration; T/P ratio: concentration ratio of tumor/plasma.

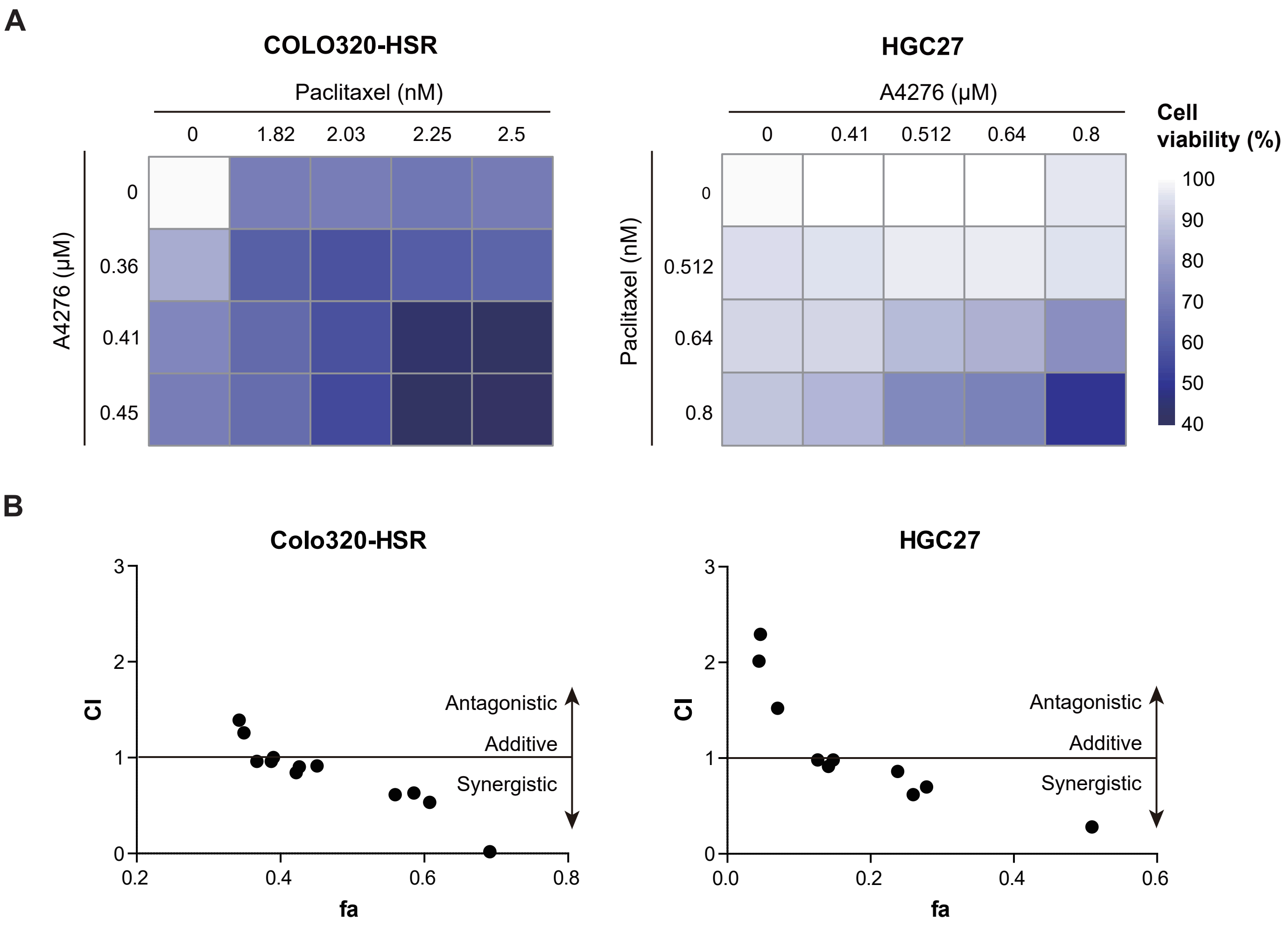


**Figure S7. The combined treatment of A4276 and paclitaxel exhibits a synergistic effect in inhibiting the growth of NAPRT-deficient cancer cell lines.**

**(A)** Heatmap showing the relative viability of the indicated cell lines after 72 h of treatment with A4276, and paclitaxel at the indicated concentrations. DMSO was used as a vehicle control. The mean values obtained from biological duplicates, each performed in technical duplicates, were used. **(B)** The Fa-CI plot (Chou-Talalay plot) indicating the effects of paclitaxel and A4276 when combined. Combination index (CI) values were calculated using the Chou-Talalay method and plotted against the fraction affected (Fa) values for those with values less than 3. CI < 1 indicates synergism, CI = 1 denotes an additive effect, and CI > 1 suggests an antagonistic effect.

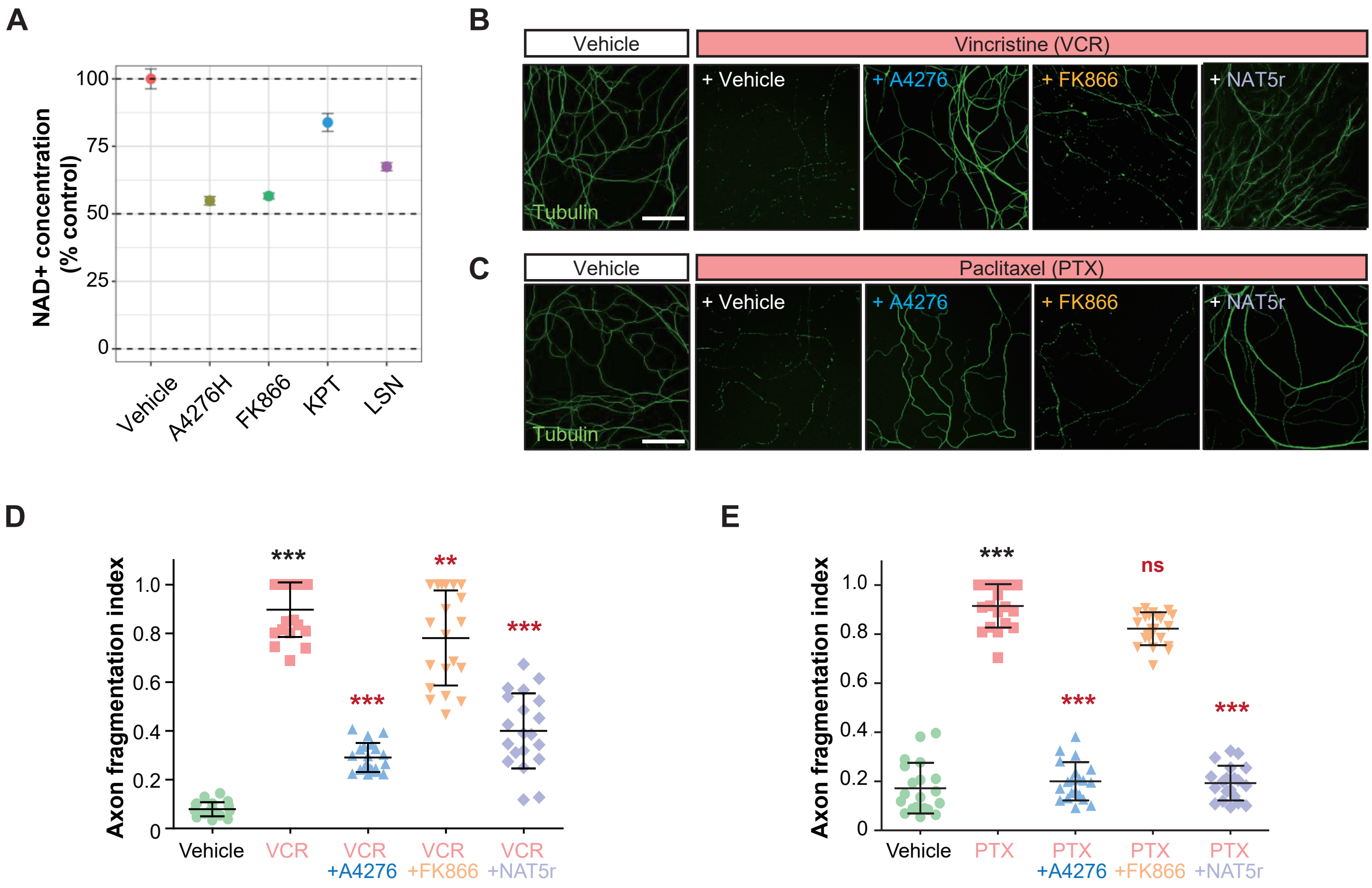


**Figure S8. A4276 prevents SARM1-dependent axon degeneration partly by decreasing the NMN-to-NAD+ ratio.**

**(A)** The concentration of NAD+ in embryos treated with different NAMPT inhibitors (10 μM) or vehicle for 48 h (n = 4 biological replicates per group). $p < 0.001$ in post hoc Tukey following one-way ANOVA for all comparisons except KPT, where $p = 0.006$, when compared to the vehicle control. Data represents mean ± SD. **(B-C)** Representative immunocytochemical images of acetylated alpha-tubulin, treated with chemotherapeutics (vincristine 40 nM or paclitaxel 100 nM) with the vehicle, A4276 (2 μM), FK866 (5 μM), or NAT5r (5 μM). **(D-E)** Quantification of axon degeneration. ***$p < 0.001$, **$p < 0.01$, and ns (not significant) in post hoc Tukey following one-way ANOVA. Black asterisks indicate comparisons to the vehicle-treated group, and red asterisks indicate comparisons to the vincristine (VCR) or paclitaxel (PTX) only group. Data represents mean ± SD (n=20).

**Table S1. Results of hERG inhibition assay for A4276 and A4276H.**

| Treatment | | Concentration (μM) | hERG cardiac potassium channel inhibition (%) |
|---|---|---|---|
| Positive control | E4031 | 10 | 87.7 ± 5.41 |
| Test compound | A4276 | 10 | 12.0 ± 5.04 |
| Positive control | E4031 | 10 | 92.8 ± 6.11 |
| Test compound | A4276H | 10 | 14.6 ± 1.84 |

| A4276 Concentration (μM) | $IC_{50}$ (μM) |
|---|---|
| 0.001, 0.01, 0.1, 1, 10, 100 | Predicted over 100 μM |

The assay was conducted at a single concentration of 10 μM (upper panel) and at six 10-fold serial dilutions ranging from 0.001 μM to 100 μM to determine the $IC_{50}$ value (lower panel). E4031 was used as the positive control.

**Table S2. SRM transitions and parameters of NMN, NAD+ and 2-chloroadenosine**

| Analytes | Q1 (m/z) | Q3 (m/z) | Declustering potential (eV) | collision energy (eV) | collision cell exit potential (eV) |
|---|---|---|---|---|---|
| NMN | 335.027 | 123.100* | 60 | 19 | 14 |
| | 335.027 | 79.900** | 60 | 79 | 10 |
| NAD+ | 664.058 | 428.000* | 80 | 37 | 10 |
| | 664.058 | 136.000** | 80 | 101 | 12 |
| 2-chloroadenosine | 302.079 | 170.000* | 60 | 29 | 10 |
| | 302.079 | 134.000** | 60 | 53 | 14 |

*: quantification transition, **: qualification transition

**Table S3. Statistics for structural data collection and refinement**.

| **Data collection** | | **Refinement** | |
|---|---|---|---|
| Protein data bank (PDB) code | 8IVU | Resolution range (Å) | 29.71 - 2.09 |
| Data collection | NAMPT/A4276 | Completeness (%) | 99.98 |
| Diffraction source | PAL/PLS BEAMLINE 5C | No. of reflections | 132220 |
| Wavelength (Å) | 1.00 | Final $R_{work}$ (%) | 20.41 |
| Temperature (K) | 100 | Final $R_{free}$ (%) | 23.13 |
| Detector | DECTRIS EIGER X 9M | No. of non-H atoms | |
| Rotation range per image (°) | 1.00 | NAMPT | 14920 |
| Total rotation range (°) | 360 | A4276 | 104 |
| Exposure time per image (s) | 0.50 | Water | 710 |
| Space group | $P2_12_12_1$ | $PO_4$ | 60 |
| *a*, *b*, *c* (Å) | 97.56, 116.69, 195.41 | Total | 15794 |
| α, β, γ (°) | 90, 90, 90 | R.m.s. deviations | |
| Mosaicity (°) | 0.09 | Bonds (Å) | 0.002 |
| Resolution range (Å) | 29.71 - 2.09 (2.15-2.09) | Angles (°) | 0.58 |
| Total No. of reflections | 1818859 | *B*-factors (Å$^2$) | |
| No. of unique reflections | 132238 | NAMPT | 46.59 |
| Completeness (%) | 99.9 (100.0) | A4276 | 41.86 |
| Redundancy | 13.8 (14.2) | Water | 45.60 |
| $\langle I/\sigma(I)\rangle$ | 8.8 (1.42) | $PO_4$ | 51.04 |
| $R_{meas}$ (%) | 20.3 (23.5) | Ramachandran plot | |
| | | Most favoured (%) | 98.11 |
| | | Allowed (%) | 1.89 |
